# “*SHANK3* deficiency alters early progenitor dynamics and reveals shared pathways with neurodegeneration”

**DOI:** 10.1038/s41380-025-03433-y

**Published:** 2026-01-31

**Authors:** Elisa Varella-Branco, Elizabeth Shephard, Victor H. C. Toledo, Igor C. Ramos, Ellen C. M. Lacerda, Laura L. M. Carvalho, Marcella A. Fiuza, Mayara Paschalidis, Claudia I. S. Costa, Ana C. S. Girardi, Ana C. V. Krepischi, Erasmo B. Casella, Guilherme Polanczyk, Karina Griesi-Oliveira, Fabio Papes, Lucas Alvizi, Gerson S. Kobayashi, Maria Rita dos Santos e Passos Bueno

**Affiliations:** 1 Centro de Pesquisa sobre o Genoma Humano e Células Tronco (CEGH-CEL), Instituto de Biociências, Universidade de São Paulo, São Paulo, Brazil; 2 Instituto de Psicologia, Universidade de São Paulo, São Paulo, Brazil; 3 Unidade de Neuropediatria do Instituto da Criança, Hospital das Clínicas da Faculdade de Medicina, Universidade de São Paulo, São Paulo, Brazil; 4 Departamento de Psiquiatria, University of São Paulo Medical School, São Paulo, Brazil; 5 Instituto de Ensino e Pesquisa Albert Einstein, Albert Einstein Hospital, São Paulo, Brazil; 6 Department of Genetics, Evolution, Microbiology and Immunology, Institute of Biology, University of Campinas, Campinas, Brazil; 7 Center for Medicinal Chemistry, University of Campinas, Campinas, Brazil; 8 Department of Cell and Developmental Biology, University College London, London, UK

**Keywords:** Genetics, Neuroscience, Autism spectrum disorders, Prognostic markers

## Abstract

Phelan-McDermid Syndrome (PMS), primarily linked to *SHANK3* haploinsufficiency, presents with complex neurodevelopmental features, including developmental regression, whose underlying mechanisms are poorly understood. This study investigated the impact of *SHANK3* disruption across multiple levels, from gene expression in patient-derived iPSC neurons to in vivo brain network activity. RNA-sequencing of iPSC-derived neurons from PMS patients with *SHANK3* disruption only (n = 9) and controls (n = 7) revealed dysregulation in differential gene expression and co-expression modules linked to cell cycle, RNA metabolism, and metabolic pathways in *SHANK3*-mutated neurons. All modules were correlated with PMS regression and enriched for genes implicated in neurodevelopmental or neurodegenerative disorders, such as autism, ADHD, and Alzheimer’s disease. At the cellular level, *SHANK3*-mutated cultures exhibited increased proliferation of neural progenitors and intermediate progenitor markers. Differentiated neurons showed reduced morphological complexity, specific changes in postsynaptic marker density and puncta size, and electrophysiological characteristics suggestive of neuronal hyperexcitability. Electroencephalography (EEG) in a PMS patient cohort (n = 20) compared to controls (n = 30) demonstrated hyperconnectivity and excessive high-frequency oscillations, suggesting altered neural network dynamics. In summary, the use of different analytical approaches suggested that *SHANK3* haploinsufficiency disrupts neurodevelopmental trajectories and revealed that regression in PMS may share common genes and pathways with neurodegeneration. We also characterized molecular and neurophysiological markers that can be useful in therapeutic protocols for PMS.

## Introduction

Synaptopathies, resulting from synaptic dysfunction, are commonly associated with autism spectrum disorders (ASD). Among them, Phelan-McDermid syndrome (PMS - OMIM#606232) stands out as the most prevalent, representing 0.05–0.7% of autistic individuals, and up to 2% of those with moderate to profound intellectual disability (ID) [1, 2]. PMS is an autosomal dominant disorder mostly characterized by developmental delay, hypotonia, ID, epilepsy, speech impairment, and an increased risk of ASD [3–6]. Importantly, when transitioning to adulthood, individuals with PMS experience notable regression in cognitive and neurological functions that is often accompanied by conditions such as bipolar disorder and psychosis [7–10]. *SHANK3* is disrupted in PMS cases due to 22q13 microdeletions of different sizes or intragenic nonsense/frameshift variants [4]. *SHANK3* variants have been associated with a wider range of neurological and behavioral conditions beyond PMS, including ASD [11], schizophrenia [12], bipolar disorder [13], and ID [14]. Besides its association with neurodevelopmental disorders, *SHANK3* expression is reduced in aged and Alzheimer’s disease brains [15], suggesting a role for this gene in neurodegeneration. Understanding the complex mechanisms by which *SHANK3* influences brain function and behavior is crucial for developing targeted therapies not only for PMS individuals, but also to a wide spectrum of neuropsychiatric and neurodegenerative conditions linked to this gene.

*SHANK3*, a member of the SHANK family, encodes a cytoskeletal scaffold protein critical for the structure and function of the postsynaptic density (PSD) at excitatory synapses, which are fundamental components of neuronal circuits [16]. The SHANK3 protein engages with numerous synaptic intracellular interactors, such as HOMER1 and proteins of the GKAP/SAPAP family. It is essential for organizing the postsynaptic density and anchoring neurotransmitter receptor complexes, including AMPA, mGlu, and NMDA glutamate receptors [16]. *SHANK3* underexpression results in significant synaptic deficiencies, alteration in the morphology and arborization of neurons in animal models and humans [17–23]. However, the precise nature and direction of changes in synaptic transmission strength (both excitatory and inhibitory) and intrinsic neuronal excitability in different model systems are still controversial [17–24]. These findings may be in part related to the use of cells with heterogeneous types of genetic mutations causing PMS. Thus, human in vitro models focusing on *SHANK3* alterations only, remain a valuable approach for clarifying *SHANK3* neuronal effects in humans and complementing insights from other models [12, 23].

Clinical EEG assessments in PMS often reveal abnormalities, including general slowing, changes in occipital dominant rhythm, and epileptiform activity [24, 25], patterns also commonly seen in ASD. EEG provides insights into interactions between brain regions and complex cortical networks, which are frequently disrupted in neurodevelopmental disorders, making it a valuable translational tool to explore cellular and synaptic imbalances. Utilizing EEG allows us to assess brain connectivity underlying neural network disruptions, helping bridge the gap between in vitro models and clinical traits in PMS.

Here, we applied an experimental framework that combined patient-derived and isogenic cellular models to address *SHANK3*-specific neurodevelopmental effects and establish associations with key clinical features of PMS. We report that *SHANK3* haploinsufficiency leads to transcriptional dysregulation of cell proliferation/division and DNA repair pathways, which correlate with seizures, apraxia of speech and regression. Additionally, *SHANK3*-mutated neural populations show increased proliferation alongside synaptic and electrophysiological abnormalities in derived neurons. We found evidence of neuronal and brain hyperconnectivity, based on in vitro and in vivo electrophysiological analyses, suggesting that network perturbations may represent a core feature of the disorder. This multi-level integration of transcriptomic, functional, and electrophysiological data provides a more comprehensive understanding of the disorder’s pathophysiology, highlighting potential molecular targets and cellular phenotypes to guide the development of future therapeutic strategies for PMS.

## Materials and methods

### Patient ascertainment

Individuals with PMS diagnosis were ascertained with the collaboration of the AFSPM (Associação Amigos e Familiares da Síndrome de Phelan-McDermid - Brazil). All participants had a positive PMS diagnosis based on genetic tests such as CGH-array or exome sequencing. For this study, we only included patients harboring deletions smaller than 0.2 Mb in the 22q13.3 region ([hg38]Chr22:50665819-50759338) or *SHANK3* sequence variants, totaling six and three cases respectively. Clinical aspects were evaluated by neurologists, and an electronic questionnaire was answered by the patients’ parents (Supplementary Table S1). This research was approved by the Ethics Committee of the Instituto de Biociências – Universidade de São Paulo (CAAE: 56459522.0.0000.5464) and this project obtained written informed consent from all participants/legal guardians.

### Psychological assessment

The Social Communication Questionnaire (SCQ) Lifetime version was used to assess patients’ social-communication difficulties, and the Vineland Adaptive Behavior Scales-3rd edition (Vineland-3) to characterize developmental levels (consistent with research in other genetic syndromes, see Yates et al. [25]. Both online questionnaires were completed by parents/caregivers for research purposes, not individual diagnosis.

### Exome sequencing

The presence of copy number variations (CNVs), single nucleotide variants (SNVs), and insertions/deletions (indels) in peripheral blood DNA was evaluated to exclude second-hit diagnoses. Exome libraries were prepared using SureSelect QXT (Agilent V6) and IDT xGen panels (V1.0/V2.0), sequenced on Illumina platforms (HiSeq 2500/NovaSeq 6000, 100-bp paired-end reads). Reported variants were selected based on a pipeline described in Supplementary Material.

### Isolation and reprogramming of PBMCs

iPSC was generated from peripheral blood mononuclear cells (PBMCs) isolated from nine patients and seven controls. Reprogramming followed Okita et al. [26], using episomal vectors (pCXLE-hOCT3/4-shp53, pCXLE-hSK, and pCXLE-hUL) with minor modifications at Human Genome and Stem Cell Research Center – University of São Paulo (HUG-CELL – USP)[27, 28]. Each sample was tested for plasmid integration, and pathogenic/likely pathogenic CNVs were analyzed. Pluripotency was confirmed by stem cell markers and directed germ-layer differentiation included mesoderm and endoderm protocols (Supplementary Fig. S1). Cell identity was using pairwise analysis of 15 tetranucleotide repeat loci and the amelogenin gender-determining marker, comparing with germinative DNA samples (Supplementary Material).

### Generation of isogenic *SHANK3*-edited cell line

To generate an isogenic control line, a sgRNA targeting exon 17 of the *SHANK3* gene (NM_001372044.2) was designed using the Synthego design tool. Approximately 4×10⁵ iPSCs derived from a healthy control participant were transfected with the sgRNA and TrueCut™ Cas9 Protein v2 (Invitrogen, cat # A36496) using the Neon™ Transfection System (Invitrogen, Catalog no. MPK5000) according to the manufacturer’s protocols. Clonal selection was performed by dilution and gene editing was confirmed by Sanger sequencing of the targeted region and subsequent analysis using the ICE CRISPR analysis tool (Synthego) (Supplementary Fig. S2). A clone harboring the desired modification was selected and expanded. This edited cell line is referred to herein as *SHANK3-/-*.

### Neural differentiation

Neural differentiation was performed on two clones for one individual and one clone for each remaining individual (Supplementary Table S2), using a modified double SMAD inhibition protocol [29]. iPSCs cultured on Matrigel in Essential 8 Medium were induced with a neural medium containing SMAD inhibitor (1 μM dorsomorphin, 10 μM SB431542, Sigma and Cayman) until a neuroepithelial layer formed (8–12 days). Cells were then transferred to polyornithine- and laminin-coated plates with neural induction medium. After rosette formation, cells were expanded in neural maintenance medium with N-2, B-27 (Thermo Fisher Scientific), and 20 ng/mL FGF2, followed by culture in neural maintenance medium without growth factors. Neurogenesis was observed around day 28, with cells re-plated and cultured for ~60 days. NSCs and neurons were characterized at days 40 and 60 by RT-qPCR and immunofluorescence.

### RNA Extraction and RT-qPCR

Total RNA from iPSCs, and 40- and 60-day neuron cultures was isolated using NucleoSpin TriPrep (Macherey-Nagel) and treated with the TURBO DNA-free Kit (Thermo Fisher Scientific) to remove genomic DNA. RNA quantity and integrity were assessed with Qubit and Bioanalyzer instruments. Triplicate analyses were performed with Fast SYBR Green PCR Master Mix (Applied Biosystems) on a QuantStudio5 system. Gene expression was normalized to *GAPDH*, *TBP* or *HPRT1* and results are presented as mean fold changes relative to a calibrator sample.

### Immunofluorescence

iPSC, NSC, and 60-day neurons were fixed with 4% ice-cold formaldehyde for 10 min. Permeabilization and blocking were performed with 10% BSA, followed by overnight incubation with primary antibodies: anti-OCT4 (ab19857); anti-SSEA4 (ab16287); anti-SOX1 (4194S); anti-MAP2 (ab183830); anti-beta III Tubulin (TUJ1) (ab78078); anti-Synapsin I (ab8); anti-HOMER1 (sc-17842). Fluorescent conjugated secondary antibodies Alexa Fluor 488 donkey anti-mouse or Alexa Fluor 594 donkey anti-mouse (Thermo Fisher Scientific, cat# A21202 and A21203, respectively), were prepared in a blocking buffer and incubated with the sample at room temperature for 90 min. Samples were counterstained with DAPI and stored in Fluoromount-G Mounting Medium (Thermo Fisher Scientific) until imaging.

### RNA library and sequencing

RNA libraries from 60-day neurons were prepared with ribosomal RNA depletion using the Zymo-Seq RiboFree Total RNA Library Kit (Zymo Research). Sequencing on the NovaSeq platform (Illumina) generated 150 bp paired end reads, which were trimmed with Trimmomatic[30] and aligned to the GRCh38.p13 genome using STAR with optimized parameters [31]. Gene read counts were summarized with RSEM, including transcript estimation via read start position distribution [32]. Quality parameters for each sample are detailed in Table S3.

### Differential expression and weighted-gene correlation network analysis

Transcript abundances were aggregated into gene-level counts using tximport (version 1.10.1), and only genes considered expressed were kept in the following analyses (gene count > 10 in at least the number of samples for the smallest group, i.e., 6 samples). Hierarchical clustering and PCA identified an outlier (control C5), which was excluded (Supplementary Fig. S3). Differential expression analysis between PMS patients and controls was conducted using DESeq2 (version 1.22.1) [33] with a significance threshold of FDR < 0.05. Co-expression analysis using WGCNA (R package) [34] constructed a signed network, assessing correlations between modules and disease status, as well as clinical features of PMS. The signed network was constructed using a power of 24, a minimum module size of 50 and a cut height of the final merge of 0.2. For each module, gene expression levels were summarized in an eigengene value which was used to assess the correlation of a module to disease status. The same strategy, using a subset including just PMS patient’s samples (*n* = 9), was employed to estimate the correlation between the modules with age, sex, and core features of PMS, including ID, speech capacity, apraxia of speech, hypotonia, seizures, ASD, ADHD, regression, and psychosis (coded as categorical variables), Vineland and SCQ scales (coded as continuous variables) (Table S1).

Module preservation was evaluated using 200 permutations comparing our data with BrainSpan fetal brain samples (RNA-Seq Gencode v10 summarized to genes, available at https://www.brainspan.org/). We have selected samples from cortical areas ranging from the ages of to 16–24 post-conception weeks. Significance of module preservation was assessed by the Z summary value, which combines multiple preservation Z statistics [35]. Functional annotation of DEGs and WGCNA modules was performed using clusterProfiler [36], DAVID (https://david-d.ncifcrf.gov/) [37], and STRING 12.0 (https://string-db.org/cgi/input.pl) [38].

### Database enrichment analysis

Genes from each module underwent modular single-set enrichment test (MSET) [39] using the entire network as the background. Enrichment analysis identified over-represented genes in each module related to specific cell types, based on mid-gestational fetal brain gene expression [40]. For module enrichment associated with neurodevelopmental and psychiatric disorders, we used curated gene sets for conditions such as Alzheimer’s disease (AD) [41], neurodegenerative disorders [42], ADHD [43], major depressive disorder (MDD) [44], bipolar disorder [45], ID [46], syndromic and non-syndromic ASD genes from SFARI database (https://gene.sfari.org/), along with other independent ASD datasets [47, 48], schizophrenia [49], macrocephaly and microcephaly genes sourced from testing panels by the University of Chicago (https://dnatesting.uchicago.edu) and the Online Mendelian Inheritance in Man database (OMIM - https://omim.org), and cross-disorder gene lists [50, 51]. MSET was also applied to assess the overlap between the modules identified in our study and modules from studies using both post-mortem brains and neurons differentiated from iPSC that were associated with PMS [52], and ASD [53–56].

### Deconvolution analysis

Transcriptomic data was submitted to cell type frequency estimation using CIBERSORTx deconvolution recommended workflow (Supplementary Methodology references). The signature matrix representing cell types of transcriptomic profiles were based on single-cell RNA-seq data across various regions of the developing human brain [57]. Cell-specific gene signatures were covering the 39 following groups: Truncated radial Glia (tRG), Dividing Radial Glia (G2/M-phase) (RG-div1), Dividing Radial Glia (S-phase) (RG-div2), Newborn Excitatory Neuron - early born, Newborn Excitatory Neuron - late born (nEN-late), Early Born Deep Layer/subplate Excitatory Neuron V1 (EN-V1-1), Ventricular Radial Glia (vRG), CGE/LGE-derived inhibitory neurons (IN-CTX-CGE1 and 2), Dividing Intermediate Progenitor Cells RG-like (IPC-div1), Intermediate Progenitor Cells RG-like (IPC-div2), Outer Radial Glia (oRG), Intermediate Progenitor Cells EN-like (IPC-nEN 1-3), Oligodendrocyte progenitor cell (OPC), Medial Ganglionic Eminence (MGE)-derived cortex inhibitory neuron (IN-CTX-MGE 1-2), caudal and lateral ganglionic eminence (CGE/LGE)-derived inhibitory neurons (IN-CTX-CGE 1-2) Striatal neurons (IN-STR), Early Born Deep Layer/subplate Excitatory Neuron Prefrontal Cortex (PFC) (EN-PFC 1-3), MGE Radial Glia (MGE-RG), MGE newborn neurons (nIN 1-5), MGE Progenitors (MGE-IPC 1-3), dividing MGE Progenitors (MGE-div), Astrocyte, early Radial Glia (RG-early) and Microglia.

### Neuronal morphology analysis

To quantify neuronal network morphology, immunofluorescence images were acquired using a Zeiss LSM800 confocal laser scanning microscope equipped with a 20x objective. Cultures were immunoassayed for the dendritic marker MAP2 (detected with Alexa Fluor 647) and counterstained with DAPI (blue channel) to visualize cell nuclei. A total of three representative images were captured per condition from independent experiments. Automated morphological analysis was performed using the NeurphologyJ plugin [58] for ImageJ/Fiji. Given the high density and overlapping nature of the neuronal networks in our cultures, which makes the tracing of individual neurites from origin to termination challenging, we implemented an analysis workflow to quantify global morphological parameters for each entire field of view. The workflow involved two main steps: (i) Structure Segmentation: Somata were identified based on the high-intensity MAP2 signal, often clustered around DAPI-stained nuclei, and neurites were segmented based on the continuous MAP2-positive signal throughout the image. (ii) Global Parameter Quantification: The plugin then calculated collective metrics for the entire image. To account for variations in cell density between images, these global metrics were subsequently normalized by the total number of identified somata in the corresponding field of view. The following normalized indices of network morphology were calculated: Average Soma Area (µm²), Normalized Neurite Area (total neurite area / soma count), Normalized Neurite Count (total neurite segments / soma count), Average Neurite Length (µm), the Attachment Point Index (total neurite-soma intersections / soma count), and the Branching Index (total neurite endpoints / soma count). Data were pooled from the acquired images, and the results presented reflect these normalized, field-average values for each condition.

### Synaptic puncta quantification

For synaptic puncta quantification, immunofluorescence images were acquired using a Zeiss LSM800 confocal laser scanning microscope equipped with a 40x objective in Z-stack acquisition mode. Cultures were immunoassayed for the presynaptic marker SYNAPSIN (SYN1) and the postsynaptic marker HOMER1. Image analysis was performed using the SynQuant plugin [59] implemented in ImageJ/Fiji. Z-stack images were processed to generate maximum intensity projections, which were subsequently analyzed with SynQuant. Individual puncta were identified by segmenting regions of interest based on fluorescence intensity and morphological parameters. To improve detection accuracy and minimize false positives, a size and shape filter was applied during analysis, retaining only puncta with an area ≥ 0.1 µm² and a roundness ≥ 0.4. The final dataset included puncta counts and spatial distribution metrics extracted from three images per condition.

### Multi-electrode array analysis

We used 24-well multi-electrode array plates (M384-tMEA-24W, Axion Biosystems) to record electrical activity from neural cells. At 50 days of neural differentiation, 2×10⁴ cells were plated per well, with 12 independent replicates per subject. Neurons were cultured in BrainPhys medium (STEMCELL) until the 60-day measurement, with medium changes twice a week. Spontaneous activity was recorded using a Maestro system and AxIS software (Axion Biosystems, version 1.0), with a 10–2.5 kHz bandwidth filter. Spike detection used an adaptive threshold of 5.5 times the standard deviation of estimated noise for each electrode. Data analysis, via the Neural Metrics Tool (Axion Biosystems, version 2.5.1), identified active electrodes with at least 5 spikes per min. Bursts were detected using an adaptive Poisson surprise algorithm and network bursts were defined by a minimum of 10 spikes in more than 25% of electrodes with an interspike interval of less than 100 ms. Only wells with bursting activity were analyzed.

### Flow cytometry

Neural cell type quantification in culture was performed using the BD Stemflow™ Human Neural Lineage Analysis Kit (BD Biosciences), which includes monoclonal antibody conjugates for neural differentiation markers: anti-SOX2 (51-9007227), anti-GFAP (51-9007228), anti-DCX (51-9007229), anti-Nestin (51-9007230), anti-Ki-67 (51-9007231), anti-SOX1 (51-9007232), and anti-CD44 (51-9007233). Experiments were conducted according to the manufacturer’s instructions, with appropriate isotype and unstained controls. At least 30,000 events were acquired per sample.

For cell cycle analysis, neurons were cultured on laminin-coated 6-well plates until 70% confluence, dissociated, fixed with 4% formaldehyde, and stained with 7-AAD (Millipore) following the manufacturer’s protocol. Cell debris and doublets were excluded based on forward and side scatter plots (FSC-A vs. SSC-A) and area versus height plots (FSC-A vs. FSC-H). 7-AAD fluorescence histograms (cell count vs. intensity) were generated, and gates were set to distinguish G0/G1, S, and G2/M phases.

Cell proliferation was assessed using the Click-iT™ Plus EdU Alexa Fluor™ 488 Flow Cytometry Assay Kit (Thermo Fisher Scientific, C10632). Approximately 1×10⁶ cells were seeded per well in 12-well plates and incubated with 10 µM EdU for 6 h. After incubation, cells were harvested, washed, fixed, and permeabilized according to the manufacturer’s protocol. Immunostaining was first performed with anti-TBR2 (Abcam, ab23345), followed by incubation with Goat anti-Rabbit IgGm Superclonal™ Secondary Antibody, Alexa Fluor™ 647 (Thermo Fisher Scientific, A27040). Subsequently, cells were stained with PE-conjugated anti-PAX6 (BD Biosciences, 561552). Flow cytometry acquisition was performed on a BD FACSymphony™ A1 Cell Analyzer and data analysis was conducted using FlowJo™ Software (v10.8, BD Life Sciences). Gating strategies were defined based on both isotype and unstained controls to determine positive cell populations.

### EEG acquisition and processing

A total of 20 patients with Phelan-McDermid Syndrome (PMS) and 30 neurotypical controls, age- and sex-matched, participated in this study. Controls scoring above the autism threshold on the Social Communication Questionnaire (SCQ; score >15) or below the typical range on the Vineland Adaptive Behavior Scales were excluded from further analyses. The neurotypical control group consisted of both siblings of PMS patients and unrelated healthy individuals.

Importantly, participants P5 and P9 from this PMS cohort also provided the samples used in the earlier iPSC and neuronal differentiation experiments. Continuous EEG was recorded for up to 5 min, with participants seated comfortably at 60 cm in front of a computer monitor while watching a silent movie of their choice. EEG recordings were acquired using a 128-channel HydroCel Geodesic Sensor Net and a Net Amps 400 amplifier (Electrical Geodesics Inc., Oregon, USA), referenced online to electrode Cz and sampled at 500 Hz. Electrode impedances were maintained below 50 kΩ.

EEG data were processed offline using the HAPPE software pipeline [60] (version 3.0, MATLAB 2022b) (Supplementary Material). Oscillatory connectivity was computed in FieldTrip software [61], with clean 2-second epochs subjected to Fast Fourier Transform (FFT) to obtain Fourier coefficients for the 1–45 Hz range at 1 Hz intervals [62, 63]. Oscillatory connectivity at each frequency was quantified using the debiased weighted phase lag index (dwPLI), reflecting phase synchronization between electrodes Vinck et al. [64]. The dwPLI was computed for each electrode pair, resulting in a 99×99 matrix per frequency for each participant, averaged across the theta (4–8 Hz), alpha (8–12 Hz), beta (12–30 Hz), and gamma (30–45 Hz) bands.

Phase connectivity analysis was conducted using Network Based Statistics (NBS) [65] to compare large-scale neural networks between PMS patients and controls in each frequency band. A primary threshold of 3.0 (p < 0.05) and 5000 permutations were applied. Significant differences in brain networks were visualized using BrainNet Viewer [66]. Age was included as a covariate in all NBS models to account for age-related connectivity differences. Whole-brain phase connectivity was also computed by averaging dwPLI across all electrode pairs for each frequency band, and comparisons between groups were made using univariate ANCOVA, covarying age.

### Statistics

Individual p-values and the number of replicates for statistical testing are provided in the corresponding figure legends. Normality tests were conducted to determine the appropriate statistical tests. Other statistical tests were applied as specified in the figure legends, and all analyses were performed using GraphPad Prism.

## Results

### Genetic and clinical characterization of the Brazilian PMS cohort

**Fig. 1:**
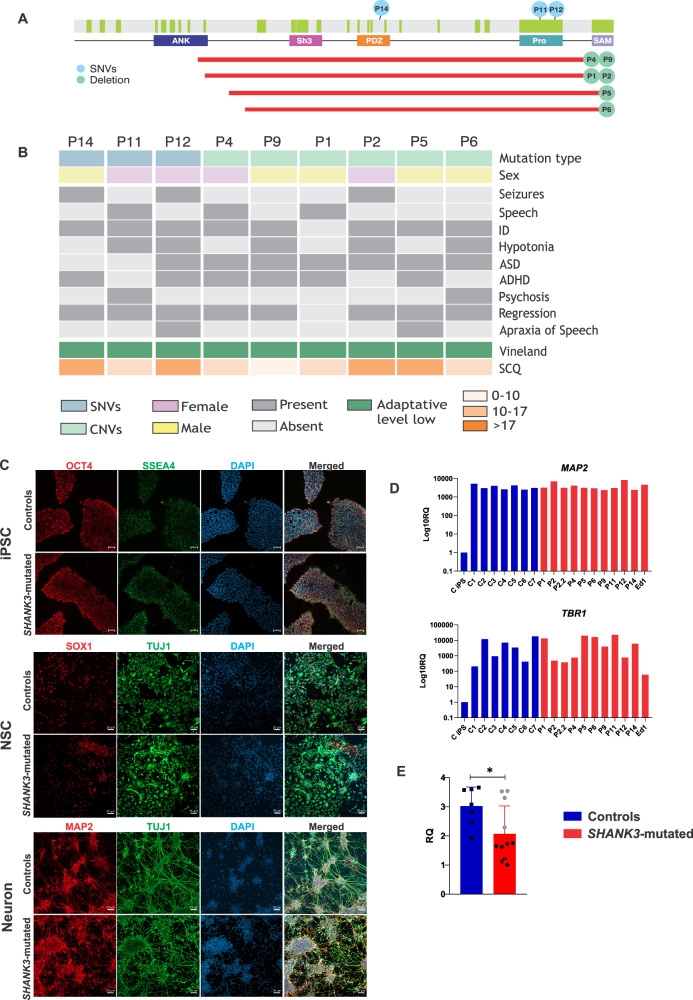
PMS Brazilian cohort characteristics and neuronal differentiation. **A** Schematic drawing of the *SHANK3* gene and the location of the Ank, Sh3 PDZ, Pro, and SAM protein domains. Exons are represented in light green. The location of causal variants of each patient as well as the size of the deletion are represented in light blue and red, respectively. **B** Clinical characteristics observed in PMS patients. The patients are ordered according to the type of the mutation. The colors are described in the legend below the figure. **C** Immunofluorescence representative images of PMS and control groups at stages of iPSC, NSC and neurons (60 days of differentiation). Immunolabeling for iPSC (OCT4 and SSEA4), NSC (SOX1 and TUJ1) and neuronal (TUJ1 and MAP2). DAPI labeling (in blue) indicates the localization of the nuclei. White scale bars measure 50 μm. **D** Relative expression of neural makers (*MAP2* and *TBR1*). P1 to P14 are differentiated PMS neurons; P2 = clone 5 and P2.2 = clone 6; Ed1 is the isogenic control *SHANK3-/-*; C1 to C7 are healthy controls; and an iPSC (C iPSC) was used as a negative control to verify the increase in the expression of neural markers after differentiation. **E** Relative Quantification (RQ) of *SHANK3* transcript levels in differentiated neurons from the *SHANK3*-mutated (n = 11 biological replicates) and control (n = 7 biological replicates) groups. Data are presented as mean ± SEM, with individual data points plotted. Within the *SHANK3-*mutated group, gray dots represent individuals with nonsense/frameshift variants, and black dots represent those with deletions. Statistical significance was determined using a two-sided Mann-Whitney U test. *p < 0.05. Data shown are from one experiment with three technical replicates averaged per biological sample.

To comprehensively assess the impact of *SHANK3* haploinsufficiency on the neural cell phenotype, we selected 9 individuals diagnosed with PMS harboring sequence variants (*n* = 3) or microdeletions (*n* = 6, size ranged from 49–110 kb) encompassing *SHANK3*, at the terminal region of 22q13.3 ([hg38] Chr22:50665819-50759338). Sequence variants predicted to cause loss of function were located at exon 21 (p. Pro1128Hisfs*167; p. Ala1227Glyfs*69; n = 2) and flanking exon 16 (splicing junction; c.2216-16_2216del: p?; n = 1) (Fig. 1A). Genomic analysis confirmed the *SHANK3* pathogenic variants and did not reveal any additional rare pathogenic/likely pathogenic variants associated with the phenotype of PMS.

The clinical assessment of the nine PMS individuals, ranging in age from four to 39 years (*mean* = 14; *SD* = 11.3), is shown in Fig. 1B and Table S1. Vineland-3 scores were between 20 and 70, falling within the low adaptation classification. On the SCQ, all patients scored higher than 15, indicative of global developmental disorder, and three of them scored over 22, suggestive of ASD (Fig. 1B). No obvious genotype-phenotype correlations were apparent upon qualitative observation (Fig. 1B).

### In vitro neural differentiation and characterization: expression of neural markers and *SHANK3*

To investigate the consequences of *SHANK3* haploinsufficiency on neural cell function, iPSCs were generated from peripheral blood cells obtained from the nine PMS individuals and seven healthy control subjects (three related; four unrelated). One unrelated control iPSC was used to generate an isogenic cell line harboring a homozygous 7 bp deletion in exon 17 of *SHANK3*(NM_001372044.2):c.2150_2156del:p.(Asn717Thrfs*3) (Supplementary Fig. S1). This isogenic line is referred to as *SHANK3-/-* throughout the study. Subsequent experiments were performed comparing PMS patient-derived lines, *SHANK3*-/- isogenic line and control-derived lines. In total, 10 iPSC lines were included in the *SHANK3* loss of function group (eight PMS, including two clones from one of the samples, and one isogenic line), hereinafter called “*SHANK3*-mutated”; and seven iPSC lines were included in the control group (Table S2). All iPSC lines were negative for vector integration, pathogenic/likely pathogenic CNVs and mycoplasma, and their identity and pluripotency phenotype were confirmed via specific molecular assessments (Supplementary Fig. S2).

*SHANK3*-mutated and control iPSCs were subjected to cortical neural induction for 60 days. At day 40, cells were positive for *SOX1* and *TUJ1*, indicating neural stem cell (NSC) identity (Fig. 1C). At 60 days of differentiation, we confirmed the presence of terminally differentiated neurons via expression of markers *MAP2*, *TUJ1, SOX1*, and *TBR1* (Figs. 1C and 1D). At this time point, *SHANK3* transcripts were confirmed to be downregulated in the *SHANK3*-mutated group (*p* = 0.02; Fig. 1E). The patients with sequence variants in *SHANK3* showed transcript levels similar to those of the controls (Gray dots - Fig. 1E). This may suggest that nonsense-mediated decay of *SHANK3* is inefficient during neuronal differentiation, as previously suggested [21].

### *SHANK3*-mutated neurons show altered gene expression in DNA repair, replication, and cell cycle

**Fig. 2:**
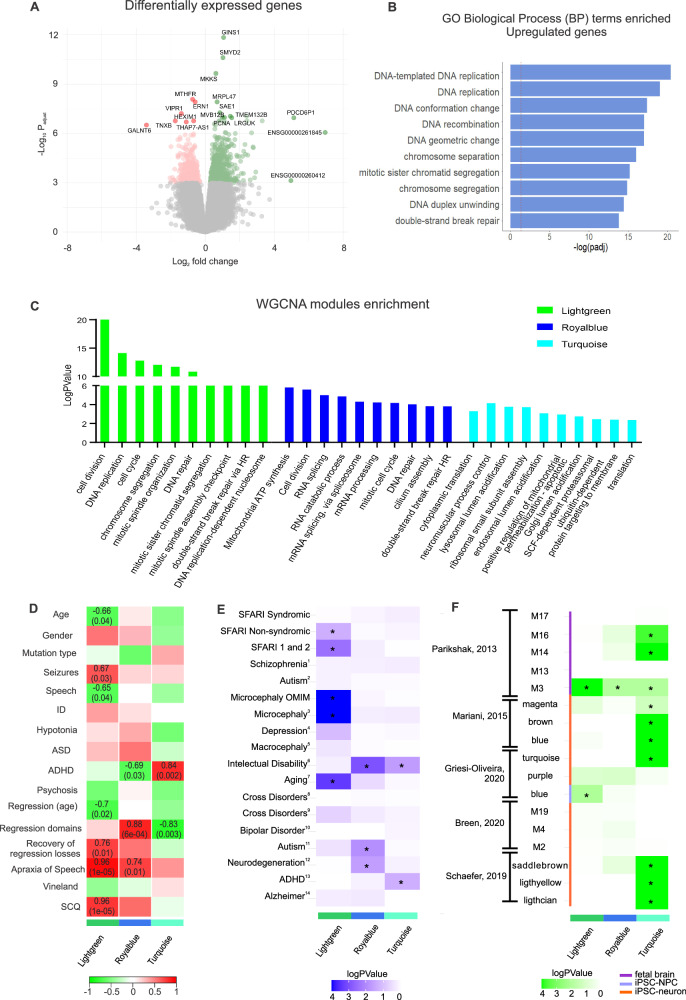
Functional annotation analysis of differentially expressed genes and WGCNA modules in *SHANK3* haploinsufficient neurons. **A** Volcano plot shows DEGs of *SHANK3* haploinsufficiency in neurons, and associated log2 fold-changes and -log10 multiple test corrected p value. Green dots represent upregulated genes and red dots represent downregulated genes. Genes highlighted are the top ones based on p-adjusted value and fold change; (**B**) Biological process top terms (FDR < 0.05) for enrichment result of upregulated genes in *SHANK3* haploinsufficiency; (**C**) Functional annotation enrichment analysis biological process terms for the three *SHANK3*-associated in Lightgreen, Royalblue and Turquoise. **D** HeatMap correlation of modules and main traits of PMS. Only the significant values are shown. Pearson coefficient is shown above and p-value is indicated in parentheses. Pathways and GO terms for each module are described on the right side. **E** Enrichment analysis using MSET to identify differential modules with PMS genes associated with neuropsychiatric disorders or conditions lists from multiple databases. ¹ Lam M et al. [49]; ² Sanders SJ et al. [47]; ^3,5^https://dnatesting.uchicago.edu; ^4^Howard DM et al. [44]; ^6^Ilyas et al., [46]; ^7^ https://genomics.senescence.info/genes/index.html; ^8^ Deciphering Developmental Disorders Study (2017); ^9^ Lee et al., [51]; ^10^ Stahl et al., [45]; ^11^ Grove et al., [48]; ^12^Orme et al., [42]; ^13^ Demontis et al., [43]; ^14^ Jansen et al., [41]. Disorders to which differential modules significantly enriched are indicated by asterisks (p value < 0.05). Heatmap colors refer to log p value. **F** Module overlap analysis comparing the PMS-associated modules identified in this study with transcriptome studies conducted either with postmortem brain samples (purple) or iPSC-derived neuronal cells (orange). All the modules that presented significant overlap PMS-associated modules (p value < 0.05) are highlighted with an asterisk. Heatmap colors refer to log p value.

To address the transcriptomic changes due to *SHANK3* haploinsufficiency, bulk RNAseq was carried out on neurons at 60 days of differentiation. A total of 903 differentially expressed genes (DEGs) were detected in *SHANK3*-mutated neurons compared to controls (*FDR-corrected p-value* < 0.05) (Table S4). In the *SHANK3*-mutated group, 553 genes were upregulated, while 350 genes were downregulated (Fig. 2A).

Enrichment analysis of these DEG lists revealed that, while no enrichment was found in the downregulated genes, the upregulated genes were enriched for categories related to cell cycle, cell division, and DNA replication (Table S5). Gene Ontology (GO) Biological Process (BP) terms included DNA recombination, DNA replication and negative regulation of cell cycle (Fig. 2B); Molecular Function (MF) terms included catalytic activity (acting on DNA) and single-stranded DNA binding; and Cellular Compartment (CC) included chromosome regions, nuclear chromosomes and condensed chromosomes. Accordingly, pathway analysis showed enrichment for Cell Cycle (KEGG: hsa04110; Reactome: R-HSA-69620), Homologous Recombination (hsa03440), and DNA Replication (hsa03030).

### Gene co-expression networks highlight RNA metabolism and translation in *SHANK3* haploinsufficiency

To further explore the findings and uncover novel relationships within the transcriptomic data, we utilized weighted gene co-expression network analysis (WGCNA) to identify genes with similar expression patterns in neurons and determine their correlation with PMS. A total of 24 modules of co-expressed genes were identified (Supplementary Fig. S4 and Table S6), most of which are moderately (2 < *z-score* < 10) or strongly (*z-score* > 10) preserved in transcriptome data from fetal brain cortices at 16–24 post conception weeks (pcw) from the BrainSpan atlas (https://www.brainspan.org/) (FigureS4). Three modules were significantly correlated with disease status and supported by protein-protein interaction (PPI) evidence: Lightgreen, Royalblue and Turquoise (PPI enrichment *p-values* < 1.0e-16). Lightgreen and Royalblue were positively correlated (*r* = 0.69; *p* = 0.002 and *r* = 0.72; *p* = 0.001), whereas Turquoise was negatively correlated with disease status (*r* = −0.57; *p* = 0.02) (Supplementary Fig. S5-S6).

The Lightgreen module is associated with the cell cycle pathway (KEGG:04110 and REAC: R-HSA-1640170), and GO terms related to cell division, DNA replication, and chromosome segregation (Fig. 2C). In turn, Royalblue and Turquoise revealed additional disease associations: Royalblue was enriched for RNA metabolism (REAC:R-HSA-8953854) and oxidative phosphorylation (KEGG:00190) pathways, and GO terms mitochondrial ATP synthesis, cell division, RNA splicing and RNA catabolic process (Fig. 2C); Turquoise, module that includes *SHANK3*, was enriched for metabolic pathways (KEGG:01100) and translation elongation pathways (REAC:R-HSA-156842), and GO terms cytoplasmic translation, neuromuscular process controlling balance, lysosomal lumen acidification and ribosomal small subunit assembly (Fig. 2C). In addition, all modules showed at least one term related to synapses process, neural maintenance and brain development (Table S7).

### Co-expression modules correlate with clinical findings in PMS

Next, we explored module correlations with the main clinical features of PMS ascertained in our cohort, as well as age, sex, and psychometric scores (Vineland and SCQ). The clinical traits selected were seizures, speech capacity, hypotonia, ADHD, psychosis and apraxia of speech. We also included traits related to regression episodes: age of first regression episode, number of domain losses, and recovery of characteristics.

The Lightgreen module showed positive correlations with PMS traits: seizures (*r* = 0.67; *p* = 0.03), recovery of regression losses (*r* = 0.76; *p* = 0.01), apraxia of speech (*r* = 0.96; *p* = 1e-05) and SCQ score (*r* = 0.96; *p* = 1e-05) (Fig. 2D). A negative correlation with age (*r* = −0.66; *p* = 0.04), speech (*r* = −0.65; *p* = 0.04) and age of first regression episode (*r* = −0.7; *p* = 0.02) (Fig. 2D). We select the top hub genes based on kME values of association in the module (*kME* > 0.8), yielding a list of 431 genes. Some top hub genes are reported in ID (26 genes), most of them related to chromosome organization (i.e. *H4C1*, *H4C2*, *H4C8* and *H4C9*). Also, two of them, *SPAST* and *KDM3B*, are associated with apraxia of speech. Interestingly, some of the top hub genes present in this module are associated with Microcephaly (*LMNB1*, *ASPM*, *KIF11*, *KIF14*, *NCAPH*, *STIL*, *KNL1* and *CENPE*). Also, we highlight genes associated with neurodegenerative disorders, *CAVIN4*, *LMNB1* and *SETMAR*.

Royalblue module showed positive correlations with number of lost domains in regression (*r* = 0.88; *p* = 6e-04) and apraxia of speech (*r* = 0.74; *p* = 0.01), and a negative correlation with ADHD (*r* = −0.69; *p* = 0.03) (Fig. 2D). Genes reported in neurodegenerative pathways are also enriched in these top genes, like Amyotrophic lateral sclerosis, Parkinson’s disease, Huntington’s disease and prion disease (*ATP5PD*, *ATP5PB*, *DNMT1*, *NDUFS1*, *NDUFS6*, *RAB8A*, *COX6C*, *HNRNPA1L2*, *HNRNPA2B1*, *HNRNPA3*, *NCBP1*, *PSMA4*, *PSMC3*, *PSMD14*, *PSMD7*, *SRSF7*, *UBE2L3*).

Finally, Turquoise was positively correlated with ADHD (*r* = 0.84; *p* = 0.002) and negatively correlated with regression domains (*r* = −0.83; *p* = 0.003) (Fig. 2D). In this module, we observed genes enriched for primary mitochondrial disease (*ATPAF2*, *NDUFS7*, *NDUFV1*, *NDUFC2, KCTD14*, *TUFM*, *COX6B1*, *COX8A*, *CYC1*, *GFER*, *MIEF2*, *MRPS2*, *PUS1* and *SDHAF1*), dystonia (*ATP1A3*, *PNKD*, *KMT2B*, *PRRT2*, *TMEM151A*) and congenital disorder of glycosylation (*ALG12*, *CCDC115*, *MPI*, *SSR4*, *SLC37A4*). Also, 31 genes are associated with intellectual disability. Moreover, these top genes are related with ribosome, metabolic pathways and oxidative phosphorylation pathways. The pathways related to neurodegenerative disorders, Huntington disease, AD and Parkinson disease, are also present in this module.

These analyses thus revealed several deregulated pathways associated with important clinical features of PMS.

### Overlap of *SHANK3* modules with genes linked to neurodevelopmental and neurodegenerative disorders

To assess the role of neurodegenerative and neurodevelopmental processes in the *SHANK3* pathology, we used Modular Single-Set Enrichment Test (MSET) on the disease-associated modules to search for overlapping genes previously implicated in neurodevelopmental, neurodegenerative disorders and aging. Genes related ASD, ID, ADHD, microcephaly (neurodevelopmental), neurodegenerative disorders and aging were enriched in the modules. (Fig. 2E). Lightgreen was enriched for nonsyndromic SFARI (ASD) genes, microcephaly genes and aging genes; Royalblue was associated with genes harboring common ASD risk variants [48], genes associated with ID [46], and with neurodegenerative disorders [42]; lastly, Turquoise was also enriched with ID [46], and ADHD genes [43] (Fig. 2E).

To gain additional insight into the relationship between the etiology of ASD and *SHANK3* pathology, we investigated whether Lightgreen, Royalblue and Turquoise overlapped with ASD-associated modules identified in previous transcriptomic analyses. Lightgreen and Royalblue overlapped with early neurodevelopment ASD modules; both overlapped with M3 from Parikshak et al. [53], an early cortical development module enriched with rare ASD variants, while Lightgreen overlapped with the blue module from Griesi-Oliveira et al. [56], an ASD neural progenitor module (Fig. 2F). Conversely, Turquoise overlapped with ASD modules associated with different stages of neurogenesis (magenta, brown and blue from Mariani et al. [55]; saddlebrown, lightyellow and lightcyan from Schafer et al [54]), in addition to turquoise from Griesi-Oliveira et al. [56], an ASD neuron module, and M14 and M16 from Parikshak et al. [53], an ASD cortical development module (Fig. 2F). Finally, no overlap was found between our disease-associated modules and those derived from a genetically heterogeneous sample of PMS individuals (i.e. harboring both point mutations/deletions affecting *SHANK3* alone, and deletions encompassing *SHANK3* and additional genes) [52] (Fig. 2F).

These results reveal that the disease-associated modules identified in *SHANK3*-mutated samples are linked to co-expressed genes implicated in neurodegeneration, ASD and other neurodevelopmental disorders.

### Cell type enrichment and deconvolution reveal deficits in radial glia and progenitors

**Fig. 3:**
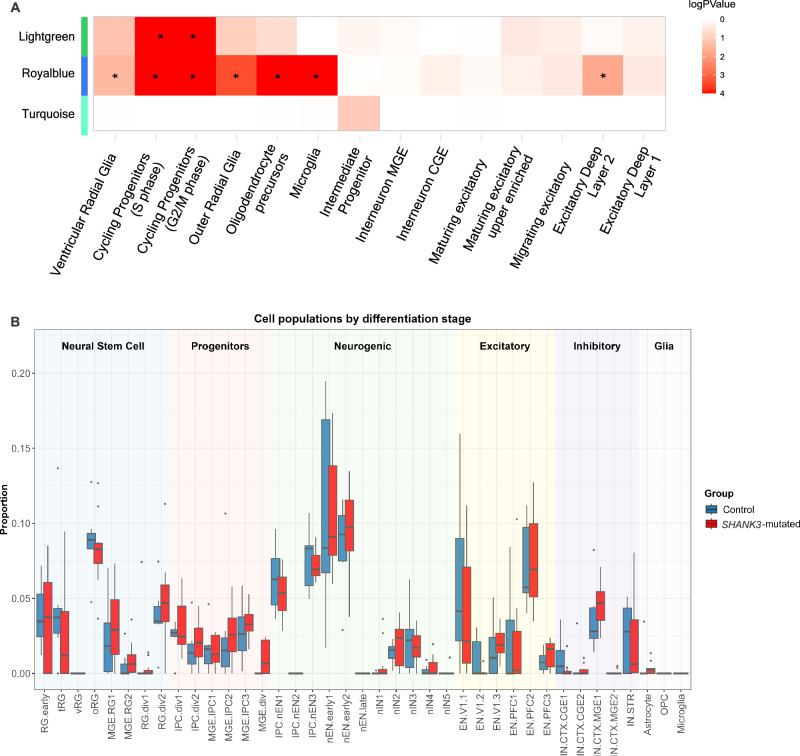
Cell enrichment in transcriptomic data. **A** Enrichment analysis using MSET using neural cell profiles. All cell types that present significant enrichment in each module (p value < 0.05) are highlighted with an asterisk. Heatmap colors refer to log p value. **B** Boxplots comparing the estimated cellular proportions between Control (blue; n = 7 biological replicates) and SHANK3-mutated (red; n = 9 biological replicates) groups, deconvoluted from the bulk RNAseq data. Cell subtypes are organized into major developmental stages (Neural Stem Cell, Progenitors, Neurogenic, Excitatory, Inhibitory, and Glia), indicated by shaded background colors. Each boxplot displays the median (center line). Individual samples are shown as dots. Statistical comparisons between groups for each cell type were performed using a two-sided Mann-Whitney U test. The resulting p-values were subsequently adjusted for multiple comparisons using the Benjamini-Hochberg False Discovery Rate (FDR) method.

Next, to ascribe cell type context to the disease-associated WGCNA modules, we used MSET to look for enrichment of specific neural lineage signatures [40]. Cell type enrichment analyses highlighted overrepresentation of markers for ventricular radial glia, cycling progenitor cells and excitatory neurons (Fig. 3A). Lightgreen and Royalblue were both enriched for cycling progenitors in the S and G2/M phases, while Royalblue was enriched for other progenitor types such as ventricular and outer radial glia, and oligodendrocyte precursor, and excitatory deep layer neurons. In contrast, Turquoise did not exhibit enrichment for any specific cell type (Fig. 3A). These results agree with the observed overlap with early neurodevelopment ASD modules for Lightgreen and Royalblue, and with the overlap with ASD modules representing different stages of neurogenesis for Turquoise. Overall, these results suggest a dysfunction in proliferative radial glia cells and excitatory neurons in the *SHANK3*-mutated group.

To estimate cell type composition from our transcriptomic data, we performed a deconvolution analysis using publicly available single-cell RNA-sequencing data from the developing human brain, encompassing 39 cell groups [57]. Both *SHANK3*-mutated and control neural populations were predominantly composed of cell types of characteristics of early neurogenesis, including radial glia, intermediate progenitors and newborn neurons. For better visualization, these cell groups were organized into six broader stages of neural differentiation: Neural Stem Cell, Progenitors, Neurogenic, Excitatory, Inhibitory and Glia (Fig. 3B). The analysis revealed a trend toward an increase in the proportion of progenitor-stage cells in the *SHANK3*-mutated group compared to controls (Fig. 3B; p = 0.6). No differences were observed between groups in the proportions of more differentiated cell types (Excitatory, Inhibitory, and Glia). We confirmed the expression of specific markers for neural progenitor, excitatory and inhibitory neurons by quantitative PCR (Supplementary Fig. S7).

### SHANK3 haploinsufficiency dysregulates early proliferative neural cells

**Fig. 4:**
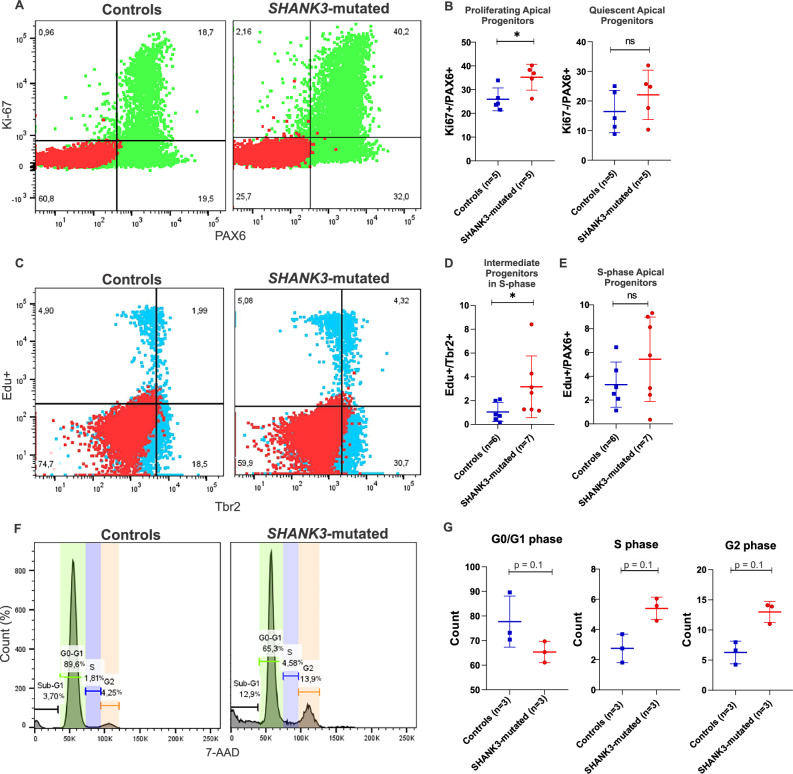
Haploinsufficiency of *SHANK3* impairs neural cell proliferation. **A** Flow cytometry of Ki-67 and PAX6; (**B**) Quantification of apical progenitor populations. The graph on the left shows the percentage of proliferating apical progenitors (Ki67 + /PAX6 + ), and the graph on the right shows quiescent apical progenitors (Ki67-/PAX6 + ). Data are presented as mean ± SEM, with individual biological replicates from Control (n = 5) and *SHANK3-*mutated (n = 5) groups plotted. Statistical significance was determined using a two-sided Mann-Whitney test. *p < 0.05; ns, not significant. **C** Representative flow cytometry dot plots showing co-staining for the S-phase marker EdU and the intermediate progenitor marker Tbr2. **D** Quantification of the percentage of S-phase intermediate progenitors (EdU + /Tbr2 + ) in Control (n = 6) and *SHANK3*-mutated (n = 7) cultures. Data are presented as mean ± SEM with individual biological replicates plotted. Statistical significance was determined using a two-sided Mann-Whitney test. *p < 0.05. **E** Quantification of the percentage of S-phase apical progenitors (EdU + /PAX6 + ) in Control (n = 6) and *SHANK3*-mutated (n = 7) cultures. Data are presented as mean ± SEM with individual biological replicates plotted. Statistical analysis was performed using a two-sided Mann-Whitney test (ns, not significant). **F** Representative histograms from flow cytometry analysis of the cell cycle distribution, assessed by 7-AAD DNA staining in Control and *SHANK3-*mutated neural cultures. Gates indicate the proportion of cells in Sub-G1, G0/G1, S, and G2/M phases. **G** Quantification of the percentage of cells in each phase of the cell cycle for Control (n = 3) and *SHANK3-*mutated (n = 3) cultures. Data are presented as mean ± SEM, with individual biological replicates plotted. Statistical analysis was performed using a two-sided Mann-Whitney test. (p = 0.1 for G0/G1, S, and G2/M phase comparisons).

WGCNA analysis suggested an increased proportion of proliferating progenitors in *SHANK3*-mutated neural cultures and highlighted dysregulation of cell cycle–related pathways. To determine which neural populations contributed to these alterations, we performed flow cytometry using a panel of neural stem and progenitor markers, including the proliferation marker Ki-67. Compared to controls, *SHANK3*-mutated cultures showed a significant increase in proliferating apical progenitor cells (PAX6 + /Ki-67 + ) (Fig. 4A–B), whereas other early progenitor populations (SOX2 + /Ki-67 and Nestin + /Ki-67) did not differ significantly (Supplementary Fig. S8). These findings suggest that apical progenitors remain actively cycling in *SHANK3*-mutated cultures.

When assessing S-phase entry using EdU incorporation, we observed an increase in TBR2+ intermediate progenitors but not in PAX6+ progenitors (Fig. 4C-E and Supplementary Fig. S9). This indicates that *SHANK3* mutation is associated with deregulation of cell cycle dynamics within specific progenitor subtypes.

Cell cycle phase distribution analysis revealed a trend toward accumulation of cells in S and G2/M phases in the *SHANK3*-mutated group, accompanied by a reduction in G0/G1 (p = 0.1) (Fig. 4F–G and Supplementary Fig. S10). These differences did not reach statistical significance, likely due to the limited sample size (n = 3 per group).

Notably we did not detect differences in the proportions of young neurons (DCX + ) or glial cells (GFAP + ) between groups at the analyzed time point (Supplementary Fig. S11). This suggests that the *SHANK3* defect primarily affects progenitor cell cycle regulation, without altering the generation of postmitotic neurons or glia under the culture conditions and differentiation window examined here.

### *SHANK3*-mutated neurons exhibit impaired neuronal functionality

**Fig. 5:**
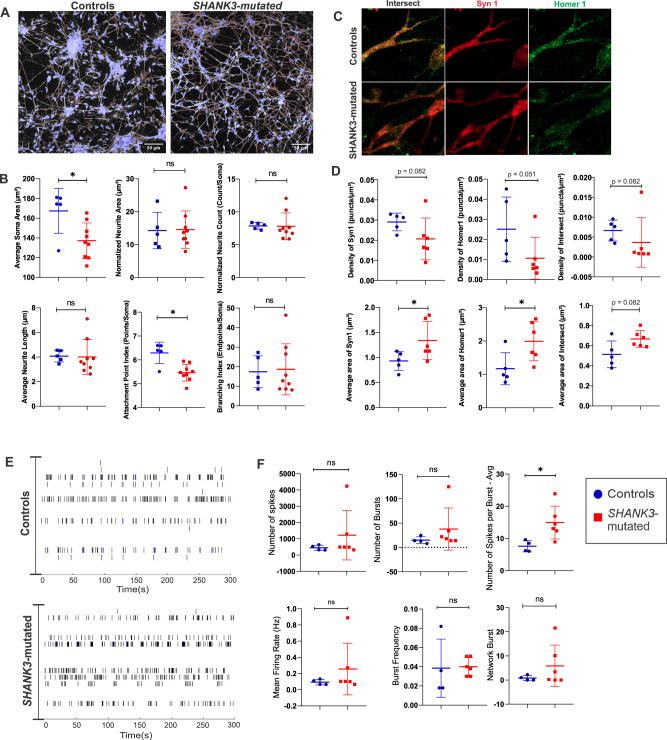
Cell types and neuronal functionality in *SHANK3* haploinsufficiency and control cell cultures. **A** Representative images of control and *SHANK3*-mutated neuronal cultures output for morphological analysis. Soma in blue, illustrating the complex network formation in both conditions. Scale bar = 50 µm. **B** Quantification of global network morphology in control and *SHANK3*-mutated cultures. All parameters represent field-of-view indices normalized by the total soma count to account for cell density. Data are presented as individual data points with mean ± SEM. Statistical analysis was performed using the Mann-Whitney test; *p < 0.05. **C** Representative images of synaptic puncta quantification in PMS and controls (Controls = 5; *SHANK3*-mutated = 5). Syn1 Synpasin 1 (Presynaptic marker - red); Homer 1 Postsynaptic marker (green); Intersect co-localization of pre and postsynaptic markers (synaptic punctas - yellow). **D** Box plots of density and area of punctas Mann-Whitney *p < 0.05. **E**) Raster plots showing electrical activity of PMS and control neurons in vitro subjected to MEA analysis (Controls = 4; *SHANK3*-mutated = 6). Each row of spikes represents an electrode. **F** Parameters obtained from MEA. Mann-Whitney test *p < 0.05.

To evaluate the morphological consequences of *SHANK3* haploinsufficiency at the neuronal network level, we conducted a comprehensive analysis of global neuronal network morphology. This phenotype was characterized by a significantly reduced average soma area (p < 0.05) and a significant reduction in the attachment point index (p < 0.05), a metric reflecting the overall density of neurite-soma connections across the network, when compared to controls (Fig. 5A-B). In contrast, other metrics of overall neurite network complexity showed no statistically significant differences between the groups.

To investigate the impact on synaptic connectivity, we analyzed the density and size of synaptic puncta by co-staining the presynaptic marker SYNAPSIN 1 (SYN1) and the postsynaptic marker HOMER 1 (Fig. 5C–D). Consistent with SHANK3’s role as a postsynaptic scaffold protein, *SHANK3*-mutated neurons displayed a reduction in the density of postsynaptic puncta (p = 0.051). We also observed that the average area of postsynaptic and presynaptic puncta was significantly larger in *SHANK3*-mutated neurons compared to controls (p < 0.05). Taken together, these data suggest a complex synaptic phenotype characterized not only by a potential disruption of the postsynaptic scaffold, but also by a potential compensatory enlargement or abnormal maturation of synaptic puncta.

Electrophysiological evaluation using multielectrode array (MEA) recordings revealed that *SHANK3*-mutated neurons exhibited an increased number of spikes per burst (p < 0.05), with no significant alterations in mean firing rate, total number of spikes, or number and frequency of bursts (Fig. 5G–H). These results suggest that *SHANK3* haploinsufficiency leads to neurons with reduced morphological complexity, impaired synaptic function, and potentially increased excitability.

### PMS patients show hyperconnected neural networks and excessive high-frequency global connectivity

Given the central role of synaptic and network dysfunction in PMS, we investigated large-scale brain connectivity patterns in PMS individuals through scalp EEG analysis. These clinical EEG data provide a neurophysiological framework to deepen the understanding of the *SHANK3* haploinsufficiency functional impact in addition to our in vitro electrophysiological neuronal analysis (Fig. 5G–H).

We included PMS patients with different mutation types: sequence variants (n = 2), small deletions <0.1 Mb (n = 4), deletions between 1–2 Mb (n = 3), deletions between 3–5 Mb (n = 5), and deletions >5.5 Mb (n = 4). Following quality control, data from 18 PMS patients (mean age = 108 months, SD = 64.3) and 26 typically developing controls (mean age = 99.9 months, SD = 58.9) were retained for analysis (Table S8).

**Fig. 6:**
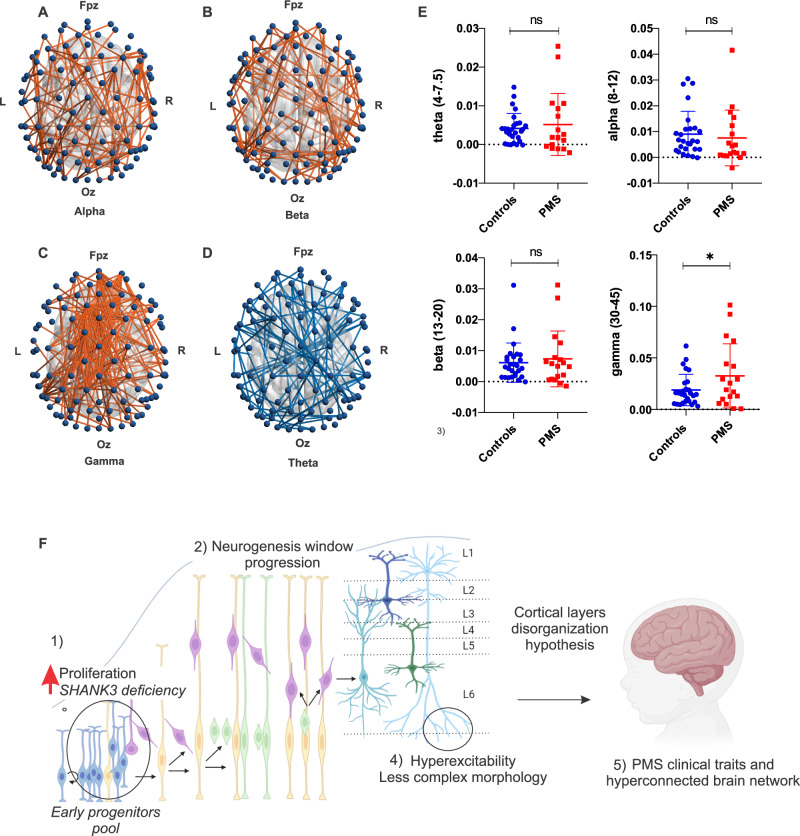
Oscillatory connectivity in PMS and proposed model of potential cortical development alterations due to SHANK3 deficiency. (**A-D**) Large-scale oscillatory networks that differed significantly between PMS and control groups based on Network-Based Statistics: (**A-C**) hyperconnected networks in PMS in the alpha, beta, and gamma frequency bands, respectively; (**D**) hypoconnected network in the theta frequency range in PMS. **E**) Whole-brain connectivity (average dwPLI across all electrode pairs) for each frequency band in PMS and controls. ANCOVA *p < 0.05. **F**) Proposed model illustrating how *SHANK3* deficiency may impact early cortical development and network function. (1) *SHANK3* deficiency leads to increased proliferation of neural progenitor cells within the early progenitor pool. (2) This disrupts the progression of the neurogenesis window, potentially impairing radial migration and proper positioning of neurons during corticogenesis. (3) These alterations may result in cortical layer disorganization. (4) Additionally, *SHANK3* deficiency affects the morphology and excitability of mature neurons, leading to reduced dendritic complexity and hyperexcitability. (5) Collectively, these cellular and structural abnormalities may contribute to impaired brain connectivity and the neuropsychiatric traits observed in PMS, including the hyperconnected functional networks identified in EEG analyses.

Network-based statistics (NBS) revealed hyperconnected large-scale networks in PMS patients compared to controls in the alpha (p = 0.003; Fig. 6A), beta (p = 0.002; Fig. 6B), and gamma (p = 0.004; Fig. 6C) frequency bands, indicating increased synchronization in these higher-frequency ranges, typically associated with excitatory activity. In contrast, NBS identified a hypoconnected large-scale network in the slow-frequency theta band (p = 0.004; Fig. 6D), suggesting reduced synchronization in networks linked to neurocognitive processes such as memory and attention.

Whole-brain connectivity analysis, measured as the average dwPLI across all electrode pairs, revealed significantly increased phase synchronization in the gamma band in PMS patients (p < 0.05), with no significant differences observed in the theta, alpha, or beta bands (Fig. 6E).

Importantly, these connectivity alterations did not correlate with mutation type within the PMS cohort.

## Discussion

In this study, we provide transcriptomic and functional insights into the effects of *SHANK3* deficiency and, for the first time, correlate this data with key clinical traits in PMS. The genetic homogeneity of our cohort allowed us to examine expression signatures resulting from *SHANK3* haploinsufficiency without the confounding effects of adjacent genes in 22q13.3 deletions. This allowed detection of associations between dysregulated co-expression modules and PMS clinical features, particularly regression. Our findings support the hypothesis that *SHANK3* plays a critical role in synapse maintenance and suggest novel roles for this gene in neurogenesis. We also demonstrated that individuals with PMS show impairment of brain connectivity, especially in high frequency waves, suggesting a hyperconnected phenotype.

Transcriptome analysis revealed a dysregulation of genes associated with cell cycle, mitotic nuclear division and DNA replication in *SHANK3*-mutated cells via different approaches (DEGs and WGCNA). While the dysregulation of cell cycle genes has been recently reported in Shank3-/- mouse brains [67], this had not been observed in previous transcriptome analysis of PMS patient-derived cells. This discrepancy is most likely due to the genetic heterogeneity of the cohort, which had individuals harboring deletions affecting additional genes [52]. In addition, based on our WGCNA PMS modules, we also confirm the relevance of terms associated with dysregulation of neuron projection, synapse regulation and axon maintenance, which is consistent with *SHANK3* canonical functions as previously reported [20, 52].

The disease-correlated modules reported here include genes crucial for neural progenitors self renewal (*CDC25A*, Lightgreen module), proliferation (*LMNB1*, Lightgreen module), neural differentiation (*LMNB1*, Lightgreen module and *DNMT1*, Royalblue module), DNA methylation (*DNMT1*, Royalblue module) and early cortical neuronal development (*CNTNAP1*, Turquoise module). Proper early neural progenitor proliferation is vital for neuron migration, cortical layer formation, and adult neurogenesis [68]. Indeed, functional analysis in this study revealed that PMS samples show a higher proportion of proliferating apical progenitors (PAX6 + ) and subsequent intermediate progenitors (TBR2 + ), suggesting a potential imbalance of early progenitor cell generation. Neural stem cells (NSCs) extracted from the subventricular zone (SVZ) of adult *Shank3* knockout mice also exhibited increased proliferation of progenitors, which underwent differentiation earlier compared to NSCs derived from wild-type individuals [69], given that *Shank3* knockout NSCs spend less time in the G0/G1 phase and more time in the G2/M phase. Additionally, a recent study showed that in *Shank3* haploinsufficiency, a smaller proportion of cells remained in a proliferative state, with a larger proportion staying in a quiescent state [70]. Overall, these results agree that *SHANK3* deficiency would affect the late phases of neurogenesis and/or the generation of mature cells rather than NSC self-renewal.

*SHANK3*, well-established as a crucial actor in synapse formation and maintenance, also appears to play roles in cell proliferation and differentiation. This may result in an increase of ratio between symmetric divisions of proliferative neural cells and asymmetric division phase. Impaired differentiation can lead to an accumulation of undifferentiated cells, suggesting a compensatory response to maintain a pool of progenitor cells [71]. These results support the hypothesis that *SHANK3* haploinsufficiency could impact neurogenesis, impairing both neuron maturation and functionality [19, 70, 72]. In this context, among the biological processes associated with PMS-related gene modules, terms such as brain neurogenesis, brain development, and layer formation in the cerebral cortex are particularly relevant. Indeed, the development of cortical structure is intimately tied to cell-cycle kinetics. Decisions about precursor cell division modes and their differentiation into neurons are influenced by the duration of different cell cycle phases, underscoring how the precise regulation of this fundamental biological process governs the sequential and coordinated generation of neurons essential for proper corticogenesis [73]. In this sense, we hypothesized that dysregulation of the cell cycle caused by *SHANK3* haploinsufficiency may lead to cortical disorganization (Fig. 6F), contributing to structural and functional brain abnormalities in PMS. Previous studies have identified ASD-related genes that play a dual role, contributing not only to synaptic function but also to the process of neurogenesis. For example, the gene *RELN*, associated with neurodevelopmental disorders, is crucial for neuronal migration and the proper organization of cortical layers. *RELN* regulates the timing of neurogenesis and migration by promoting the fate of radial glia progenitors while inhibiting premature neuronal differentiation [74]. Additionally, *POGZ*, a high-confidence ASD risk gene, plays a similar role in neurodevelopment, particularly in regulating the balance between the proliferation and differentiation of NSCs. *POGZ* knockdown results in a higher proportion of NSCs and diminished neuronal differentiation [75], which also disrupts neuronal migration to the cortical plate (largely due to impaired differentiation) leading to disorganization of lower cortical layers, a defect that persists into adulthood.

Among the disease-associated genes identified through the correlation between co-expressed genes and specific traits in PMS, we highlight those linked to aging and neurodegeneration, which are respectively related to cell cycle regulation, potentially affecting the long-term maintenance of neurogenic capacity (Lightgreen module), and mitochondrial dysfunction, such as oxidative stress (Royalblue and Turquoise modules). These types of brain cellular dysfunction have been previously associated with the above-mentioned conditions: aging [76, 77] and neurodegeneration [78]. Importantly, genes associated with neurodegeneration (such as *APP*, *PSEN1*, and *HTT*) also play key roles in neurogenesis [79–81], suggesting that dysregulated genes in neurodevelopmental disorders may confer increased risk for neurodegeneration. Indeed, individuals with ASD have a higher risk of developing Alzheimer’s disease (AD) and other forms of dementia [82]. Supporting this notion, the module associated with the age of regression episodes in PMS (Lightgreen) is enriched for genes involved in cell cycle maintenance, a critical process in corticogenesis. Notably, some adult PMS patients have been reported to show progressive clinical deterioration, characterized by rapid motor and cognitive decline, which may suggest early-onset dementia [83–88]. Additionally, neurological MRIs have revealed central brain atrophy and more recent findings report altered concentrations of tau and amyloid-beta in the cerebrospinal fluid of individuals with PMS [89].

Our data confirm that *SHANK3*-mutated neurons exhibit reduced morphological complexity and a density reduction of postsynaptic punctas, consistent with previous reports [22, 89]. A key finding of our study is the co-occurrence of these structural deficits with neuronal hyperexcitability, a seemingly paradoxical phenomenon. This outcome is consistent with emerging models of neurodevelopmental disorders that propose distinct effects on synaptic integrity versus intrinsic neuronal properties [90]. The observed reduction in HOMER1 puncta density likely reflects SHANK3 disruption within the postsynaptic scaffolding network, rather than a loss of synapses, as previously reported [16]. As a central PSD organizer, SHANK3 directly interacts with and stabilizes multiple synaptic proteins, including HOMER1, GKAP, and GluA1, and its deficiency has been shown to reduce their levels [16]. In this context, hyperexcitability may arise from compensatory mechanisms or direct impacts on intrinsic excitability, such as the dysregulation of *Ih* currents mediated by HCN channels, which can increase neuronal firing rates independently of synaptic structure [91]. Furthermore, our finding that both pre- and postsynaptic puncta exhibit increased area in *SHANK3*-mutated neurons suggests compensatory enlargement or abnormal maturation, pointing to a complex remodeling process rather than a simple reduction in connectivity. Importantly, our study strengthens that these changes arise directly from *SHANK3* deficiency and may help reconcile conflicting findings in the literature, which likely reflect the genetic heterogeneity of PMS variants across different studies [17, 18, 20–22].

In PMS individuals, we identified a pattern of network hyperconnectivity in higher-frequency bands, particularly gamma, alongside reduced connectivity in the theta band. Both hyperconnectivity and increased gamma activity were not correlated with PMS mutation type, suggesting that *SHANK3* haploinsufficiency is a key factor in these impairments. While acknowledging the significant differences in scale and biological complexity between whole-brain EEG in patients and cellular-level MEA recordings, this macro-scale hyperconnectivity is consistent with the neuronal hyperexcitability we observed in vitro and in animal models [23, 92]. Notably, enhanced gamma oscillatory power has been reported in other ASD-related syndromes [93–95] and is also associated with language skills in ASD [96]. Brain connectivity abnormalities could be a consequence of altered proliferation and functional capacity of neural cells, as seen in other neurodevelopmental conditions [97]. Hyperconnectivity at higher frequencies supports the observed hyperexcitability and may contribute to sensory and behavioral impairments, commonly seen in ASD and ADHD [63]. Furthermore, the reduced theta oscillations, which are crucial in attention, memory, and inhibition [98], could be linked to the cognitive impairments in PMS. Future research establishing correlations between neurons in vitro signatures, in vivo EEG patterns, and clinical outcomes would be highly valuable for advancing translational tools or future biomarker candidates.

In summary, this study includes transcriptomic, in vitro functional and in vivo brain activity analyses. Our findings suggest that *SHANK3* haploinsufficiency disrupts fundamental processes related to early neural progenitor proliferation and neurogenesis, potentially impacting broader neurodevelopmental trajectories. These observations lend support to the hypothesis that altered neurodevelopment in PMS could contribute to vulnerabilities later in life, such as regression, which may share mechanistic overlaps with early-onset dementia. Further research is essential to explore strategies for mitigating such significant clinical features.

## Supplementary information

Suppementary Methods

Supplementary document

Table S1. Clinical aspects and genetic characteristics of PMS patients.

Table S4. List of differentially expressed genes (DEGs) Shank3-mutated X Controls

Table S5. Enrichment analysis of DEGs up-regulated in SHANK3-mutated neurons

Table S6. WGCNA summary. kME table modules.

Table S7. Enriched Terms at PMS correlated modules

## Data Availability

Sequencing data have been deposited in the National Center for Biotechnology Information Gene Expression Omnibus (GSE281741).
