# Supplementary material for: “SHANK3 deficiency alters early progenitor dynamics and reveals shared pathways with neurodegeneration”
Source: Mol Psychiatry. 2026 Jan 31;31(6):3033–48. doi: 10.1038/s41380-025-03433-y (PMC13190290; doi:10.1038/s41380-025-03433-y)
Supplement: Supplementary file 1 — Suppementary Methods [file 41380_2025_3433_MOESM1_ESM.pdf]

**Supplementary Methods**

# "*SHANK3* deficiency alters early progenitor dynamics and reveals shared pathways with neurodegeneration"

Elisa Varella-Branco[1*]; Elizabeth Shephard[2], Victor H. C. de Toledo[1]; Igor C. Ramos[1]; Ellen C. M. Lacerda[1]; Laura L. M. Carvalho[1]; Marcella A. Fiuza[1]; Mayara Paschalidis[1]; Claudia I. S. Costa[1], Ana C. S. Girardi[1]; Ana C. V. Krepischi[1]; Erasmo B. Casella[3]; Guilherme Polanczyk[4]; Karina Griesi-Oliveira[5]; Fabio Papes[6,7]; Lucas Alvizi[8]; Gerson S. Kobayashi[1]; Maria Rita Passos-Bueno[1]

**Author affiliations:**

[1]Centro de Pesquisa sobre o Genoma Humano e Células Tronco (CEGH-CEL), Instituto de Biociências, Universidade de São Paulo, São Paulo, Brazil

[2] Instituto de Psicologia, Universidade de São Paulo, Brazil.

[3]Unidade de Neuropediatria do Instituto da Criança, Hospital das Clínicas da Faculdade de Medicina, Universidade de São Paulo, São Paulo, Brazil.

[4]Departamento de Psiquiatria, University of São Paulo Medical School, Brazil

[5]Instituto de Ensino e Pesquisa Albert Einstein, Albert Einstein Hospital, São Paulo, Brazil

[6]Department of Genetics, Evolution, Microbiology and Immunology, Institute of Biology, University of Campinas, Brazil

[7]Center for Medicinal Chemistry, University of Campinas, Brazil

[8]Department of Cell and Developmental Biology, University College London, UK.

Correspondence to: PhD. Maria Rita dos Santos e Passos-Bueno

Full address: Genetic development laboratory, Department of Genetic and Evolutionary Biology, Institute of Biosciences, University of São Paulo. Rua do Matão 277, Cidade Universitária. Zip code: 05508-090. São Paulo, Brazil.

E-mail: passos@ib.usp.br

*Patient ascertainment*

Individuals with PMS diagnosis were ascertained with the collaboration of the AFSPM (Associação Amigos e Familiares da Síndrome de Phelan-McDermid - Brazil). All participants had a positive PMS diagnosis based on genetic tests such as CGH-array or exome sequencing. For this study, we only included patients that had deletions smaller than 0.2Mb in the 22q13.3 region ([hg38]Chr22:50665819-50759338) or *SHANK3* sequence variants, totaling six and three cases respectively. Clinical aspects were evaluated by neurologists, and an electronic questionnaire was answered by the patients' parents (Table S1).

This research adheres to the ethical standards established in the Declaration of Helsinki (1964) and subsequent revisions, as well as Resolution 466/2012 of the Brazilian National Health Council. Approved by the Ethics Committee of the Instituto de Biociências – Universidade de São Paulo (CAAE: 56459522.0.0000.5464), this project obtained written informed consent from all participants/legal guardians.

*Psychological assessment*

The Social Communication Questionnaire (SCQ) Lifetime version is a screening tool to identify signs of Autism Spectrum Disorder (ASD) in individuals aged 4 and above. We used it to assess patients' social-communication difficulties related to autism. The Vineland Adaptive Behavior Scales-3rd edition (Vineland-3) is a standardized instrument that measures adaptive behavior and functioning in individuals from birth to adulthood. We used it to characterize patients' developmental level (consistent with research in other genetic syndromes, see (1)

Both psychological scales were administered as online questionnaires and were completed by parents and caregivers. In this study, these assessments were utilized to characterize the psychological condition of all patients for research purposes and were not intended for individual diagnosis.

*Exome sequencing*

The presence of copy number variations (CNVs), single nucleotide variants (SNVs), and insertions/deletions (indels) was evaluated in peripheral blood DNA from patients to exclude possible second-hit diagnoses. Exome sequencing libraries were generated using: SureSelect QXT Target Enrichment for Illumina Multiplexed Sequencing - V6 by Agilent Technologies, IDT – xGen Exome Research Panel V1.0, and IDT – xGen Exome

Research Panel V2.0 by Integrated DNA Technologies (IDT). The libraries underwent sequencing using either an Illumina HiSeq 2500 sequencer or an Illumina NovaSeq 6000 sequencer, producing paired-end reads of approximately 100 base pairs. Sequence alignments to the human genome reference (UCSC hg38) were executed using the Burrows-Wheeler Aligner (2). Data processing and variant calling were carried out with Picard (version 2.18.7) (3) and the Genome Analysis Toolkit package (GATK v4.0.9.0) (4). SNVs and indels within exons or within 50 base pairs of splicing junctions were identified in individual samples using GATK HaplotypeCaller and jointly genotyped using GATK GenotypeGVCFs. Following variant quality score recalibration, the called variants underwent annotation using ANNOVAR v.2016Feb01. CNVs larger than 5 kb were analyzed using the NextGENe software (V2.4.2.3 – SoftGenetics), following the parameters recommended by the developer.

Only rare CNVs, SNVs, and indels (frequency less than 1% based on data from populational databases (5–9) were analyzed. Rare missense, loss of function SNVs (including stopgain, stoploss, frameshift insertion, frameshift deletion, and splicing variants affecting canonical sites) and CNVs were classified according to the ACMG (American College of Medical Genetics and Genomics) guidelines (9). Only CNVs and SNVs classified as pathogenic or likely pathogenic associated with the phenotype of PMS were considered.

*Isolation and reprogramming of PBMCs*

Erythroblast expansion was conducted using peripheral blood mononuclear cells (PBMCs) isolated with SepMate tubes (STEMCELL Technologies) and Ficoll (Sigma) gradient from nine patients and seven controls. Reprogramming followed the Okita et al., 2013 (10) methodology, based on nucleofection (Amaxa Nucleofector Instrument, Lonza) of non-integrative episomal vectors (pCXLE-hOCT3/4-shp53, pCXLE-hSK, and pCXLE-hUL – Addgene #27077, #27078, and #27080, respectively), which was established with minor modifications at the Human Genome and Stem Cell Research Center – University of São Paulo (HUG-CELL – USP) (11,12). iPSC colonies with typical morphology were then transferred to Matrigel (BD Biosciences) coated plates and fed with Essential 8 medium (Life Technologies) supplemented with 0.1 mg/mL Normocin (Invivogen).

All iPSC colonies were tested for plasmid integration via end-point PCR. The presence of pathogenic/likely pathogenic CNVs were investigated using SALSA MLPA Probemix

P070-B3 and P036-E3 Subtelomeres mix (MRC-Holland) and chromosomal microarray analysis by array-CGH (Agilent 180K platform). Cell pluripotency was evaluated through the expression of stem cell markers (*OCT4*, *NANOG* and *SSEA4*) prior to differentiation. Cell identity was confirmed using pairwise analysis of 15 tetranucleotide repeat loci and the amelogenin gender-determining marker (AmpFLSTR™ Identifiler™ Plus; Thermo Fisher Scientific), comparing with germinative DNA samples (peripheral blood/oral swab).

Directed germ-layer differentiation was carried out in RPMI 1640 medium (Thermo Fisher Scientific) containing 1× B-27 without vitamin A (Thermo Fisher Scientific) and 0.1 mg/mL Normocin (Invivogen), supplemented with 5 μM CHIR99021 (Tocris) for two days (mesoderm differentiation), or supplemented with 50 ng/mL Wnt-3a (R&D Systems) and 100 ng/mL Activin A (Thermo Fisher Scientific) for three days, after which Wnt-3a was withdrawn for two days (endoderm differentiation). Neural differentiation implies ectoderm conversion (Figure 1C), so we did not generate ectoderm in this initial characterization of pluripotency. Cells were routinely tested for mycoplasma with the MycoScope PCR Detection kit (Genlantis) and the 4C Reagent Mycoalert plus (Lonza).

*Generation of isogenic SHANK3-edited cell line*

For CRISPR-Cas9 gene editing, the single guide RNA (sgRNA) for the exon 17 of *SHANK3* was designed on the Synthego platform, and transfected with the Neon$^{TM}$ Transfection System (Invitrogen; Catalog no. MPK5000) and TrueCut$^{TM}$ Cas9 Protein v2 (Invitrogen; cat # A36496), according to the manufacturer's instructions. In brief, $4x10^5$ iPSCs from a healthy control were rinsed with PBS and resuspended in buffer R, mixed with the sgRNA, Cas9, buffer R and subsequently electroporated (1200V, 20ms, 2 pulses). The transfected cells were plated onto a Matrigel-coated 6-well plate, expanded and cryopreserved. Clonal selection was performed by diluting the cell suspension and plating one cell per well of a 96 well plate. Cells were cultivated with CloneR (StemCell Technologies, 05888_C) and were assessed for gene editing with Sanger sequencing and the ICE Crispr analysis tool (https://ice.synthego.com/#/) (Figure S2).

*Neural differentiation*

Neural differentiation was carried out in two clones for one individual, and one clone per each of the remaining individuals (Table S2), with a modified protocol based on the double inhibition of the SMAD pathway (13). In summary, iPSC was cultured on Matrigel

(Corning)-coated dishes in Essential 8 Medium (Thermo Fisher Scientific) until 80–100% confluent. At this point, cells were cultured in an induction neural medium containing SMAD inhibitor (1 μM dorsomorphin and 10 μM SB431542 - Sigma and Cayman, respectively). Over a span of eight to 12 days, a uniform neuroepithelial layer emerged, and cells were harvested and seeded onto polyornithine (Sigma) and laminin-coated (Thermo Fisher Scientific) plates with neural induction medium supplemented with SMAD inhibitors. Following the appearance of rosettes, the cells were cultured for four days in neurobasal medium supplemented with N-2, B-27 (both from Thermo Fisher Scientific), and 20 ng/mL FGF2 (PeproTech) to facilitate the expansion of neural stem cells (NSC). The cells were maintained in Neurobasal medium (Thermo Fisher Scientific) with N-2 and B-27, without the addition of growth factors. After substantial neurogenesis had occurred (around day 28), the cells were re-plated using Accutase (Thermo Fisher Scientific) on polyornithine and laminin-coated plates and cultured for approximately 60 days. NSC and neuronal populations were characterized via RT-qPCR and immunofluorescence for neural differentiation markers at 40 days and 60 days.

*RNA Extraction and RT-qPCR*

Total RNA from iPSCs, 40- and 60-day neuron cultures was isolated using the NucleoSpin TriPrep (Macherey-Nagel) and treated with TURBO DNA-free Kit (Thermo Fisher Scientific) to remove genomic DNA. The quantity and integrity for each RNA sample was measured using Qubit (Thermo Fisher Scientific) and 2100 Bioanalyzer (Agilent) instruments. Primer pairs were designed on Primer-BLAST and housekeeping genes (*GAPDH* and *TBP*) were adopted from the literature (12). Each sample was analyzed in triplicate with the use of Fast SYBR Green PCR Master Mix (Applied Biosystems) according to the recommendations of the manufacturer. The reactions were run in a QuantStudio5 Real-Time PCR system (Applied Biosystems). The expression of each gene was normalized to *GAPDH*, *TBP or HPRT1* housekeeping genes, and the results are shown as the mean fold change of the normalized gene expression relative to a calibrator sample.

*Immunofluorescence*

iPSC, NSC and 60-day neurons were fixed with ice-cold 4% formaldehyde for 10 minutes. Permeabilization and blocking were performed simultaneously with 10% BSA, followed by overnight incubation with one of the primary antibodies: anti-OCT4

(ab19857); anti-SSEA4 (ab16287); anti-SOX1 (4194S); anti-MAP2 (ab183830); anti-beta III Tubulin (TUJ1) (ab78078); anti-Synapsin I (ab8); anti-Homer1 (sc-17842). Fluorescent-conjugated secondary antibodies, Alexa Fluor 488 donkey anti-mouse or Alexa Fluor 594 donkey anti-mouse (Thermo Fisher Scientific, cat# A21202 and A21203, respectively), were prepared in a blocking buffer and incubated with the sample at room temperature for 90 minutes. Samples were counterstained with DAPI and stored in Fluoromount-G Mounting Medium (Thermo Fisher Scientific) until imaging.

*RNA library and sequencing*

RNA libraries from 60-day neuron cultures were prepared with ribosomal RNA depletion using the Zymo-Seq RiboFree Total RNA Library Kit (Zymo Research), following the manufacturer's instructions. Sequencing was performed at NovaSeq platform (Illumina) to generate 150 bp paired-end sequences. The resulting reads were trimmed and quality-filtered using Trimmomatic (14) and aligned to the reference human genome (GRCh38.p13 primary assembly) using STAR with reduced spurious junctions ('--outFilterType BySJout') and extra alignment score for junctions of 1 ('--sjdbScore 1'), generating BAM files aligned to the transcriptome (15). Read counts per gene were summarized using RSEM, calculating read start position distribution ('--estimate-rspd') to better estimate transcripts (16). The summary of quality parameters for each sample is described in Table S3.

*Differential expression and weighted-gene correlation network analysis*

*tximport* (version 1.10.1) was employed to aggregate transcript abundances into gene-level counts. Only genes considered expressed were kept in the following analyses (gene count > 10 in at least the number of samples for the smallest group, i.e., 6 samples). Hierarchical clustering and principal component analysis revealed that one control (C5) was an outlier, and therefore it was excluded from the comparison (Figure S3). Differential expression analysis between PMS individuals and controls were performed using DESeq2 (version 1.22.1), considering a significance level threshold of False Discovery Rate (FDR) < 0.05 (17).

Co-expression analysis was performed with transcriptome data using the weighted correlation gene network analysis (WGCNA) package from R (18). The signed network was constructed using a power of 24, a minimum module size of 50 and a cut height of the final merge of 0.2. For each module, gene expression levels were summarized in an

eigengene value which was used to assess the correlation of a module to disease status. The same strategy, using a subset including just PMS patients samples (n=9), was employed to estimate the correlation between the modules with age, sex, and core features of PMS, including ID, speech capacity, apraxia of speech, hypotonia, seizures, ASD, ADHD, regression, and psychosis (coded as categorical variables), Vineland and SCQ scales (coded as continuous variables) (Table S1).

Module-preservation analysis was conducted using the modulePreservation function from WGCNA using 200 permutations, comparing our data with BrainSpan fetal brain samples (RNA-Seq Gencode v10 summarized to genes, available at https://www.brainspan.org/). We have selected samples from cortical areas ranging from the ages of to 16 to 24 post-conception weeks. Significance of module preservation was assessed by the Z summary value, which combines multiple preservation Z statistics (doi:10.1371/journal.pcbi.1001057).

Functional annotation analysis of DEGs and modules obtained by WGCNA was performed using clusterProfiler (19), and Database for Annotation, Visualization and Integrated Discovery 6.8 (DAVID - https://david-d.ncifcrf.gov/) (20). Protein–protein enrichment test was performed using STRING 12.0 (https://string-db.org/cgi/input.pl) (21).

*Database enrichment analysis*

Genes from each distinct module underwent a modular single-set enrichment test (MSET) (22), utilizing the entire network as the background. MSET performs permutations to create random sets of genes to correct the statistical power, increasing the gene set enrichment accuracy. To identify the over-representation of genes within each specific module related to distinct cell types, we conducted enrichment analysis using a list of genes expressed by cell types from samples of mid-gestational developing fetal brain (23). For module enrichment associated with neurodevelopmental and psychiatric disorders, we used curated gene sets for conditions such as Alzheimer's disease (AD) (24), ADHD (25), major depressive disorder (MDD) (26), bipolar disorder (27), ID (28), syndromic and non-syndromic ASD genes from SFARI database (https://gene.sfari.org/), along with other independent ASD datasets (29,30), schizophrenia (31), macrocephaly and microcephaly genes sourced from testing panels by the University of Chicago (https://dnatesting.uchicago.edu) and the Online Mendelian Inheritance in Man database (OMIM - https://omim.org), and cross-disorder gene lists (32,33).

Additionally, we employed MSET analysis to assess the overlap between the modules identified in our study and modules from studies using both post-mortem brains and neurons differentiated from iPSC that were associated to PMS (34), and ASD (35–38).

*Deconvolution analysis*

Transcriptomic data was submitted to cell type frequency estimation using CIBERSORTx deconvolution (51). The signature matrix representing cell types transcriptomic profiles were based on single-cell RNA-seq data across various regions of the developing human brain (39). Cell-specific gene signatures were covering the 39 following groups: Truncated radial Glia (tRG), Dividing Radial Glia (G2/M-phase) (RG-div1), Dividing Radial Glia (S-phase) (RG-div2), Newborn Excitatory Neuron - early born, Newborn Excitatory Neuron - late born (nEN-late), Early Born Deep Layer/subplate Excitatory Neuron V1 (EN-V1-1), Ventricular Radial Glia (vRG), CGE/LGE-derived inhibitory neurons (IN-CTX-CGE1 and 2), Dividing Intermediate Progenitor Cells RG-like (IPC-div1), Intermediate Progenitor Cells RG-like (IPC-div2), Outer Radial Glia (oRG), Intermediate Progenitor Cells EN-like (IPC-nEN 1-3), Oligodendrocyte progenitor cell (OPC), Medial Ganglionic Eminence (MGE)-derived cortex inhibitory neuron (IN-CTX-MGE 1-2), caudal and lateral ganglionic eminence (CGE/LGE)-derived inhibitory neurons (IN-CTX-CGE 1-2) Striatal neurons (IN-STR), Early Born Deep Layer/subplate Excitatory Neuron Prefrontal Cortex (PFC) (EN-PFC 1-3), MGE Radial Glia (MGE-RG), MGE newborn neurons (nIN 1-5), MGE Progenitors (MGE-IPC 1-3), dividing MGE Progenitors (MGE-div), Astrocyte, early Radial Glia (RG-early) and Microglia.

*Neuronal morphology analysis*

To quantify neuronal network morphology, immunofluorescence images were acquired using a Zeiss LSM800 confocal laser scanning microscope equipped with a 20x objective. Cultures were immunostained for the dendritic marker MAP2 (detected with Alexa Fluor 647) and counterstained with DAPI (blue channel) to visualize cell nuclei. A total of three representative images were captured per condition from independent experiments. Automated morphological analysis was performed using the NeurphologyJ plugin (40) for ImageJ/Fiji. Given the high density and overlapping nature of the neuronal networks in our cultures, which makes the tracing of individual neurites from origin to termination challenging, we implemented an analysis workflow to quantify global morphological parameters for each entire field of view. The workflow involved two main steps: (i)

Structure Segmentation: Somata were identified based on the high-intensity MAP2 signal, often clustered around DAPI-stained nuclei, and neurites were segmented based on the continuous MAP2-positive signal throughout the image. (ii) Global Parameter Quantification: The plugin then calculated collective metrics for the entire image. To account for variations in cell density between images, these global metrics were subsequently normalized by the total number of identified somata in the corresponding field of view.

The following normalized indices of network morphology were calculated: Average Soma Area ($\mu m^2$), Normalized Neurite Area (total neurite area / soma count), Normalized Neurite Count (total neurite segments / soma count), Average Neurite Length (μm), the Attachment Point Index (total neurite-soma intersections / soma count), and the Branching Index (total neurite endpoints / soma count). Data were pooled from the acquired images, and the results presented reflect these normalized, field-average values for each condition.

*Synaptic puncta quantification*

For synaptic puncta quantification, immunofluorescence images were acquired using a Zeiss LSM800 confocal laser scanning microscope equipped with a 40x objective in Z-stack acquisition mode. Cultures were immunostained for the presynaptic marker SYNAPSIN (SYN1) and the postsynaptic marker HOMER1. Image analysis was performed using the SynQuant plugin (41) implemented in ImageJ/Fiji. Z-stack images were processed to generate maximum intensity projections, which were subsequently analyzed with SynQuant. Individual puncta were identified by segmenting regions of interest based on fluorescence intensity and morphological parameters. To improve detection accuracy and minimize false positives, a size and shape filter was applied during analysis, retaining only puncta with an area $\geq 0.1$ $\mu m^2$ and a roundness $\geq 0.4$. The final dataset included puncta counts and spatial distribution metrics extracted from three images per condition.

*Multi-electrode array analysis*

We used 24-well multi-electrode array plates (M384-tMEA-24W) from Axion Biosystems to record electrical activity reads from neural cells. We plated $2x10^4$ cells onto each well at 50 days of neural differentiation, and we made 12 independent replicates (wells) per subject.

Neurons were cultured in BrainPhys medium until the measurement at 60 days, with medium changes every other day. Spontaneous network activity recordings were performed using a Maestro system and the AxIS software (Axion Biosystems; version 1.0), applying a bandwidth filter from 10 Hz to 2.5 kHz. Spike detection was computed with an adaptive threshold of 5.5 times the standard deviation of the estimated noise for each electrode. Plates were left untouched in the Maestro instrument for 5 min prior to recording, which lasted for 3 minutes.

Data analysis employed the Neural Metrics Tool (Axion Biosystems; version 2.5.1), under the condition that an electrode was deemed active if at least 5 spikes occurred within 1 min. Bursts were identified in the data recorded from each individual electrode using an adaptive Poisson surprise algorithm. Network bursts were identified for each well, using a nonadaptive algorithm requiring a minimum of 10 spikes occurring in more than 25% of electrodes with a maximum interspike interval of 100 ms. Only the wells that exhibited bursting activity were included in this analysis. The mean firing rate for a subject was calculated across active electrodes in all wells for that subject.

*Flow cytometry*

Neural cell type quantification in culture was performed using the BD Stemflow™ Human Neural Lineage Analysis Kit (BD Biosciences), which includes monoclonal antibody conjugates for neural differentiation markers: anti-SOX2 (51-9007227), anti-GFAP (51-9007228), anti-DCX (51-9007229), anti-Nestin (51-9007230), anti-Ki-67 (51-9007231), anti-SOX1 (51-9007232), and anti-CD44 (51-9007233). Experiments were conducted according to the manufacturer's instructions, with appropriate isotype and unstained controls. At least 30,000 events were acquired per sample.

For cell cycle analysis, neurons were cultured on laminin-coated 6-well plates until 70% confluence, dissociated, fixed with 4% formaldehyde, and stained with 7-AAD (Millipore) following the manufacturer's protocol. Cell debris and doublets were excluded based on forward and side scatter plots (FSC-A vs. SSC-A) and area versus height plots (FSC-A vs. FSC-H). 7-AAD fluorescence histograms (cell count vs. intensity) were generated, and gates were set to distinguish G0/G1, S, and G2/M phases.

Cell proliferation was assessed using the Click-iT™ Plus EdU Alexa Fluor™ 488 Flow Cytometry Assay Kit (Thermo Fisher Scientific, C10632). Approximately $1\times10^6$ cells were seeded per well in 12-well plates and incubated with 10 µM EdU for 6 hours. After incubation, cells were harvested, washed, fixed, and permeabilized according to the

manufacturer's protocol. Immunostaining was first performed with anti-TBR2 (Abcam, ab23345), followed by incubation with Goat anti-Rabbit IgGm Superclonal™ Secondary Antibody, Alexa Fluor™ 647 (Thermo Fisher Scientific, A27040). Subsequently, cells were stained with PE-conjugated anti-PAX6 (BD Biosciences, 561552). Flow cytometry acquisition was performed on a BD FACSymphony™ A1 Cell Analyzer and data analysis was conducted using FlowJo™ Software (v10.8, BD Life Sciences). Gating strategies were defined based on both isotype and unstained controls to determine positive cell populations.

*EEG acquisition and processing*

Participants for EEG were recruited via AFSPM with caregiver consent and presented a molecular genetic diagnostic for PMS. Two of them are also included for previously mentioned functional neuron differentiation (P5 and P9). The neurotypical control group involved some siblings of PMS patients and nonrelatives. In total we collected EEG from 20 patients and 30 controls, paired by age and sex. Symptoms of ASD in both groups were assessed by the parent-report SCQ and developmental ability by the parent-report Vineland (described above). Controls that scored above threshold for autism on the SCQ ($\geq$15) or below the typical range of scores on the Vineland were excluded from further analyses.

Continuous EEG was collected for up to 5 min. Participants were seated in a comfortable chair approximately 60 cm in front of a computer monitor and viewed a silent movie of their choice during, a common practice in individuals with neurodevelopmental disorders and intellectual disability (42). EEG was recorded using a 128-channel hydrocel geodesic sensor net and a Net Amps 400 amplifier (Electrical Geodesics Inc., Oregon, USA). Data were referenced online to electrode Cz and sampled at 500Hz. Electrode impedances were kept below 50 kU wherever possible.

EEG data were processed offline using the software HAPPE (Harvard Automated Processing Pipeline for Electroencephalography, (43) version 3.0 run within the MATLAB 2022b environment (Mathworks, Natick, USA). HAPPE is an automated processing pipeline optimized for developmental populations (43,44). Preprocessing in HAPPE involved: (1) removal of 29 rim channels that are usually contaminated by excessive artifacts, (2) removal of 50Hz electrical line noise using the Cleanline method from EEGlab, (3) identification of channels contaminated by excessive noise, (4) correction of muscular and ocular artifacts using a wavelet thresholding method, (5)

filtering using 0.5 high-pass and 50Hz low-pass Butterworth filters, (6) segmentation into 2-second epochs, (7) rejection of segments with residual artifacts, defined as those with amplitudes +/-200 microvolts, (8) interpolation of channels removed earlier in the processing pipeline, (9) re-referencing to the average reference.

Oscillatory connectivity was computed in FieldTrip software (45) following the methods we have used previously (42,46). The clean 2-second epochs were subjected to Fast Fourier Transform with a 10% Hanning window taper to obtain Fourier coefficients for the 1–45Hz range at 1Hz intervals. Oscillatory connectivity at each 1Hz frequency step was quantified by the debiased weighted phase lag index (dwPLI), which reflects the degree of phase synchronization between oscillatory signals at different electrodes (see Vinck et al., 2011 (47) for mathematical formula). The dwPLI was computed between each pair of electrodes across epochs, resulting in one 99x99 matrix per frequency step per participant, where each matrix element holds the dwPLI value (phase synchronization) between each pair of electrodes. Matrices were averaged across frequency steps in the theta (4–8Hz), alpha (8–12Hz), beta (12-30Hz) and gamma (30-45Hz) frequency bands. The resulting connectivity matrices (four per participant) were used in analysis.

Analysis of phase connectivity was conducted in two ways. First, we examined differences in large-scale neural networks between PMS patients and controls using Network Based Statistics (NBS) (48). NBS is a non-parametric statistical analysis method which identifies brain networks, defined as topologically connected clusters of nodes (electrodes) based on the strength of their edges (dwPLI values), that differ significantly between groups or conditions while controlling multiple comparisons. NBS first computes a test-statistic (t- or F-value) for each connection in the matrix, applies a primary threshold to each connection to isolate those with suprathreshold values, identifies topologically connected components (brain networks) among suprathreshold connections, and finally ascribes a p-value to identified networks via permutation testing. NBS models were constructed to test for differences in oscillatory neural networks between PMS patients and control groups in each frequency range separately. A primary threshold of 3.0 (equivalent to $p < .05$) and 5,000 permutations were used in all models. Brain networks that differed significantly between groups were visualized using BrainNet Viewer (49). Age was included as a covariate in all NBS given age-related differences in oscillatory connectivity (50). Second, whole-brain phase connectivity, defined as the average dwPLI across all 99 electrode pairs in the connectivity matrices, was computed

for each frequency band (theta, alpha, beta, gamma) and compared between PMS and control groups using univariate ANCOVA while covarying age (one ANCOVA model per frequency band).

*Statistics*

Individual p-values and the number of replicates for statistical testing are provided in the corresponding figure legends. Normality tests were conducted to determine the appropriate statistical tests. For t-tests, data were assumed to be normal. Other statistical tests were applied as specified in the figure legends, and all analyses were performed using GraphPad Prism. Statistical analyses related to EEG data are described above.

**References**

1. Yates JRW, MacLean C, Higgins JNP, Humphrey A, Le Maréchal K, Clifford M, et al. The tuberous sclerosis 2000 study: Presentation initial assessments and implications for diagnosis and management. Arch Dis Child. 2011 Nov;96(11):1020–5.
2. Li H, Durbin R. Fast and accurate short read alignment with Burrows-Wheeler transform. Bioinformatics. 2009 Jul;25(14):1754–60.
3. Depristo MA, Banks E, Poplin R, Garimella K V., Maguire JR, Hartl C, et al. A framework for variation discovery and genotyping using next-generation DNA sequencing data. Nat Genet. 2011 May;43(5):491–501.
4. Poplin R, Ruano-Rubio V, DePristo MA, Fennell TJ, Carneiro MO, Van der Auwera GA, et al. Scaling accurate genetic variant discovery to tens of thousands of samples [Internet]. 2017. Available from: http://biorxiv.org/lookup/doi/10.1101/201178
5. Karczewski KJ, Francioli LC, Tiao G, Cummings BB, Alföldi J, Wang Q, et al. The mutational constraint spectrum quantified from variation in 141,456 humans , Genome Aggregation Database Consortium. Nature [Internet]. 2020 [cited 2021 Dec 28];581:19. Available from: https://doi.org/10.1038/s41586-020-2308-7
6. Naslavsky MS, Scliar MO, Yamamoto GL, Wang JYT, Zverinova S, Karp T, et al. Whole-genome sequencing of 1,171 elderly admixed individuals from São Paulo, Brazil. Nat Commun. 2022 Dec 1;13(1).
7. MacDonald JR, Ziman R, Yuen RKC, Feuk L, Scherer SW. The Database of Genomic Variants: A curated collection of structural variation in the human genome. Nucleic Acids Res. 2014 Jan 1;42(D1).
8. Richards S, Aziz N, Bale S, Bick D, Das S, Gastier-Foster J, et al. Standards and guidelines for the interpretation of sequence variants: A joint consensus recommendation of the American College of Medical Genetics and Genomics and the Association for Molecular Pathology. Genetics in Medicine. 2015 May 8;17(5):405–24.
9. Riggs ER, Andersen EF, Cherry AM, Kantarci S, Kearney H, Patel A, et al. Technical standards for the interpretation and reporting of constitutional copy-number variants: a joint consensus recommendation of the American College of Medical Genetics and

Genomics (ACMG) and the Clinical Genome Resource (ClinGen). Available from: https://doi.org/10.1038/s41436-

40. Okita K, Yamakawa T, Matsumura Y, Sato Y, Amano N, Watanabe A, et al. An efficient nonviral method to generate integration-free human-induced pluripotent stem cells from cord blood and peripheral blood cells. Stem Cells. 2013 Mar;31(3):458–66.
41. Griesi-Oliveira K, Acab A, Gupta A, Sunaga D, Chailangkarn T, Nicol X, et al. Modeling non-syndromic autism and the impact of TRPC6 disruption in human neurons. Mol Psychiatry [Internet]. 2014 [cited 2024 Jan 7];20:1350–65. Available from: http://www.ingenuity.
42. Miller EE, Kobayashi GS, Musso CM, Allen M, Ishiy FAA, De Caires LC, et al. EIF4A3 deficient human iPSCs and mouse models demonstrate neural crest defects that underlie Richieri-Costa-Pereira syndrome. Hum Mol Genet. 2017;26(12):2177–91.
43. Shi Y, Kirwan P, Livesey FJ. Directed differentiation of human pluripotent stem cells to cerebral cortex neurons and neural networks. Nat Protoc. 2012;7(10):1836–46.
44. Bolger AM, Lohse M, Usadel B. Trimmomatic: a flexible trimmer for Illumina sequence data. Bioinformatics [Internet]. 2014 Aug 1 [cited 2024 Apr 5];30(15):2114–20. Available from: https://dx.doi.org/10.1093/bioinformatics/btu170
45. Dobin A, Davis CA, Schlesinger F, Drenkow J, Zaleski C, Jha S, et al. STAR: ultrafast universal RNA-seq aligner. Bioinformatics [Internet]. 2013 Jan [cited 2024 Apr 5];29(1):15. Available from: /pmc/articles/PMC3530905/
46. Li B, Dewey CN. RSEM: Accurate transcript quantification from RNA-Seq data with or without a reference genome. BMC Bioinformatics [Internet]. 2011 Aug 4 [cited 2024 Apr 5];12(1):1–16. Available from: https://bmcbioinformatics.biomedcentral.com/articles/10.1186/1471-2105-12-323
47. Love MI, Huber W, Anders S. Moderated estimation of fold change and dispersion for RNA-seq data with DESeq2. Genome Biol [Internet]. 2014 Dec 5 [cited 2024 Apr 5];15(12):1–21. Available from: https://genomebiology.biomedcentral.com/articles/10.1186/s13059-014-0550-8
48. Langfelder P, Horvath S. WGCNA: An R package for weighted correlation network analysis. BMC Bioinformatics. 2008;9.
49. Yu G, Wang LG, Han Y, He QY. ClusterProfiler: An R package for comparing biological themes among gene clusters. OMICS [Internet]. 2012 [cited 2024 Apr 5];16(5):284–7. Available from: http://bioconductor.org/packages/release/bioc/html/clusterProfiler.html
50. Sherman BT, Hao M, Qiu J, Jiao X, Baseler MW, Lane HC, et al. DAVID: a web server for functional enrichment analysis and functional annotation of gene lists (2021 update). Nucleic Acids Res. 2022 Jul 5;50(W1):W216–21.
51. Szklarczyk D, Kirsch R, Koutrouli M, Nastou K, Mehryary F, Hachilif R, et al. The STRING database in 2023: protein-protein association networks and functional enrichment analyses for any sequenced genome of interest. Nucleic Acids Res. 2023 Jan 6;51(1 D):D638–46.
52. Eisinger BE, Saul MC, Driessen TM, Gammie SC. Development of a versatile enrichment analysis tool reveals associations between the maternal brain and mental health disorders, including autism. BMC Neurosci [Internet]. 2013 Nov 19 [cited 2024 Apr 5];14(1):1–15.

Available from: https://bmcneurosci.biomedcentral.com/articles/10.1186/1471-2202-14-147

23. Polioudakis D, De La Torre-Ubieta L, Langerman J, Gerstein MB, Plath K, Geschwind Correspondence DH. A Single-Cell Transcriptomic Atlas of Human Neocortical Development during Mid-gestation In Brief. Neuron [Internet]. 2019 [cited 2023 Oct 26];103:785–801. Available from: https://doi.org/10.1016/j.neuron.2019.06.011

24. Jansen IE, Savage JE, Watanabe K, Bryois J, Williams DM, Steinberg S, et al. Genome-wide meta-analysis identifies new loci and functional pathways influencing Alzheimer's disease risk. Nat Genet [Internet]. 2019 Mar 1 [cited 2024 Apr 5];51(3):404–13. Available from: https://pubmed.ncbi.nlm.nih.gov/30617256/

25. Demontis D, Walters RK, Martin J, Mattheisen M, Als TD, Agerbo E, et al. Discovery of the first genome-wide significant risk loci for attention deficit/hyperactivity disorder. Nat Genet [Internet]. 2019 Jan 1 [cited 2024 Apr 5];51(1):63–75. Available from: https://pubmed.ncbi.nlm.nih.gov/30478444/

26. Howard DM, Adams MJ, Clarke TK, Hafferty JD, Gibson J, Shirali M, et al. Genome-wide meta-analysis of depression identifies 102 independent variants and highlights the importance of the prefrontal brain regions. Nat Neurosci [Internet]. 2019 Mar 1 [cited 2024 Apr 5];22(3):343–52. Available from: https://pubmed.ncbi.nlm.nih.gov/30718901/

27. Stahl EA, Breen G, Forstner AJ, McQuillin A, Ripke S, Trubetskoy V, et al. Genome-wide association study identifies 30 loci associated with bipolar disorder. Nat Genet [Internet]. 2019 May 1 [cited 2024 Apr 5];51(5):793–803. Available from: https://pubmed.ncbi.nlm.nih.gov/31043756/

28. Ilyas M, Mir A, Efthymiou S, Houlden H. The genetics of intellectual disability: Advancing technology and gene editing. F1000Res [Internet]. 2019 [cited 2024 Apr 5];8. Available from: https://pubmed.ncbi.nlm.nih.gov/31984132/

29. Sanders SJ, He X, Willsey AJ, Devlin B, Roeder K, State MW, et al. Insights into Autism Spectrum Disorder Genomic Architecture and Biology from 71 Risk Loci Article Insights into Autism Spectrum Disorder Genomic Architecture and Biology from 71 Risk Loci. Neuron [Internet]. 2015;87(6):1215–33. Available from: http://dx.doi.org/10.1016/j.neuron.2015.09.016

30. Grove J, Ripke S, Als TD, Mattheisen M, Walters RK, Won H, et al. Identification of common genetic risk variants for autism spectrum disorder. Nat Genet. 2019;

31. Lam M, Chen CY, Li Z, Martin AR, Bryois J, Ma X, et al. Comparative genetic architectures of schizophrenia in East Asian and European populations. Nature Genetics 2019 51:12 [Internet]. 2019 Nov 18 [cited 2024 Apr 5];51(12):1670–8. Available from: https://www.nature.com/articles/s41588-019-0512-x

32. Lee PH, Anttila V, Won H, Feng YCA, Rosenthal J, Zhu Z, et al. Genomic Relationships, Novel Loci, and Pleiotropic Mechanisms across Eight Psychiatric Disorders. Cell [Internet]. 2019 Dec 12 [cited 2020 Jun 14];179(7):1469-1482.e11. Available from: https://linkinghub.elsevier.com/retrieve/pii/S0092867419312760

33. McRae JF, Clayton S, Fitzgerald TW, Kaplanis J, Prigmore E, Rajan D, et al. Prevalence and architecture of de novo mutations in developmental disorders. Nature. 2017 Feb 23;542(7642):433–8.

34. Breen MS, Browne A, Hoffman GE, Stathopoulos S, Brennand K, Buxbaum JD, et al. Transcriptional signatures of participant-derived neural progenitor cells and neurons implicate altered Wnt signaling in Phelan-McDermid syndrome and autism. Available from: https://doi.org/10.1186/s13229-020-00355-0

35. Parikshak NN, Luo R, Zhang A, Won H, Lowe JK, Chandran V, et al. XIntegrative functional genomic analyses implicate specific molecular pathways and circuits in autism. Cell [Internet]. 2013;155(5):1008. Available from: http://dx.doi.org/10.1016/j.cell.2013.10.031

36. Schafer ST, Paquola ACM, Stern S, Gosselin D, Ku M, Pena M, et al. Pathological priming causes developmental gene network heterochronicity in autistic subject-derived neurons. Nat Neurosci [Internet]. 2019;22(2):243–55. Available from: http://dx.doi.org/10.1038/s41593-018-0295-x

37. Mariani J, Coppola G, Zhang P, Pelphrey KA, Howe R, Vaccarino FM, et al. Glutamate Neuron Differentiation in Autism FOXG1-Dependent Dysregulation of GABA / Glutamate. Cell [Internet]. 2015;162(2):375–90. Available from: http://dx.doi.org/10.1016/j.cell.2015.06.034

38. Griesi-Oliveira K, Fogo MS, Pinto BGG, Alves AY, Suzuki AM, Morales AG, et al. Transcriptome of iPSC-derived neuronal cells reveals a module of co-expressed genes consistently associated with autism spectrum disorder. Mol Psychiatry [Internet]. 2021 [cited 2020 Feb 18];26(5):1589–605. Available from: https://doi.org/10.1038/s41380-020-0669-9

39. Eze UC, Bhaduri A, Haeussler M, Nowakowski TJ, Kriegstein AR. Single-cell atlas of early human brain development highlights heterogeneity of human neuroepithelial cells and early radial glia. Nat Neurosci [Internet]. 2021;24(4):584–94. Available from: http://dx.doi.org/10.1038/s41593-020-00794-1

40. Ho SY, Chao CY, Huang HL, Chiu TW, Charoenkwan P, Hwang E. NeurphologyJ: An automatic neuronal morphology quantification method and its application in pharmacological discovery. BMC Bioinformatics. 2011 Jun 8;12.

41. Wang Y, Wang C, Ranefall P, Broussard GJ, Wang Y, Shi G, et al. SynQuant: an automatic tool to quantify synapses from microscopy images. [cited 2024 Jan 7]; Available from: https://academic.oup.com/bioinformatics/article/36/5/1599/5584198

42. Shephard E, McEwen FS, Earnest T, Friedrich N, Mörtl I, Liang H, et al. Oscillatory neural network alterations in young people with tuberous sclerosis complex and associations with co-occurring symptoms of autism spectrum disorder and attention-deficit/hyperactivity disorder. Cortex. 2022;146:50–65.

43. Gabard-Durnam LJ, Leal ASM, Wilkinson CL, Levin AR. The harvard automated processing pipeline for electroencephalography (HAPPE): Standardized processing software for developmental and high-artifact data. Front Neurosci. 2018;12.

44. Monachino AD, Lopez KL, Pierce LJ, Gabard-Durnam LJ. The HAPPE plus Event-Related (HAPPE+ER) software: A standardized preprocessing pipeline for event-related potential analyses. Dev Cogn Neurosci. 2022 Oct 1;57.

45. Oostenveld R, Fries P, Maris E, Schoffelen JM. FieldTrip: Open source software for advanced analysis of MEG, EEG, and invasive electrophysiological data. Comput Intell Neurosci. 2011;2011.

46. Shephard E, Tye C, Ashwood KL, Azadi B, Johnson MH, Charman T, et al. Oscillatory neural networks underlying resting-state, attentional control and social cognition task conditions in children with ASD, ADHD and ASD+ADHD. Cortex. 2019 Aug 1;117:96–110.
47. Vinck M, Oostenveld R, Van Wingerden M, Battaglia F, Pennartz CMA. An improved index of phase-synchronization for electrophysiological data in the presence of volume-conduction, noise and sample-size bias. Neuroimage. 2011 Apr 15;55(4):1548–65.
48. Zalesky A, Fornito A, Bullmore ET. Network-based statistic: Identifying differences in brain networks. Neuroimage. 2010 Dec;53(4):1197–207.
49. Xia M, Wang J, He Y. BrainNet Viewer: A Network Visualization Tool for Human Brain Connectomics. PLoS One. 2013 Jul 4;8(7).
50. Designed Research; P RSJU, Performed Research; P FRJU. The development of neural synchrony reflects late maturation and restructuring of functional networks in humans [Internet]. Vol. 106, PNAS. 2009. Available from: www.pnas.org/cgi/content/full/0900390106/DCSupplemental.www.pnas.orgcgidoi10.1073pnas.0900390106
51. Steen CB, Liu CL, Alizadeh AA, Newman AM. Profiling Cell Type Abundance and Expression in Bulk Tissues with CIBERSORTx. Methods Mol Biol. 2020;2117:135-157. doi: 10.1007/978-1-0716-0301-7_7. PMID: 31960376; PMCID: PMC7695353.
