# Supplementary material for: “SHANK3 deficiency alters early progenitor dynamics and reveals shared pathways with neurodegeneration”
Source: Mol Psychiatry. 2026 Jan 31;31(6):3033–48. doi: 10.1038/s41380-025-03433-y (PMC13190290; doi:10.1038/s41380-025-03433-y)
Supplement: Supplementary file 2 — Supplementary document [file 41380_2025_3433_MOESM2_ESM.pdf]

"SHANK3 deficiency alters early progenitor dynamics and reveals shared pathways with neurodegeneration"

Elisa Varella-Branco[1]*; Elizabeth Shephard[2], Victor H. C. de Toledo[1]; Igor C. Ramos[1]; Ellen C. M. Lacerda[1]; Laura L. M. Carvalho[1]; Marcella A. Fiuza[1]; Mayara Paschalidis[1]; Claudia I. S. Costa[1]; Ana C. S. Girardi[1]; Ana C. V. Krepischi[1]; Erasmo B. Casella[3]; Guilherme Polanczyk[4]; Karina Griesi-Oliveira[5]; Fabio Papes[6,7]; Lucas Alvizi[8]; Gerson S. Kobayashi[1]; Maria Rita Passos-Bueno[1]

Supplementary Figures

Figure S1. Pluripotency characteristics of the iPSC lines. (A) Representative images of immunofluorescence from control (n=5) and SHANK3-mutated cells (n=9). OCT3/4 (red) and SSEA4 (green) was used as pluripotency markers. DAPI (blue) nuclei marker. Scale bar = 50 µm. (B) Relative mRNA expression of OCT3 and NANOG used as pluripotency markers. Peripheral blood cell (PBMC) was used as a negative control. (C) The genomes of iPSC lines are free of reprogramming plasmids; P: positive control; product size for OriP positive reaction: 544 bp; product size for EBNA-1 positive reaction: 666 bp; N: negative control. (D) Differentiation capacity was confirmed by evaluation of the mRNA expression of markers of mesoderm and ectoderm. Relative mRNA expression of TBXT, GCS and HAND2 was used as mesoderm markers; SOX17, FOXA2 and CRCR4 was used as endoderm markers. Control: iPSC lineage.

Figure S2. Generation of isogenic SHANK3-mutated lineage. Schematics for CRISPR/Cas-9 genome editing. We designed a sgRNA targeting SHANK3 gene at exon 17. ICE Crispr analysis tool using sanger sequence as input showed a deletion of 7 bp in homozygous.

Figure S3. Quality control from RNA-seq data of samples. (A) Hierarchical clustering analysis of samples based on gene expression data. Sample C5 is noticeably distant from the other samples, indicating it is an outlier and leading to its exclusion from further analysis. (B) Principal Component Analysis (PCA) showing the distribution of samples based on their gene expression profiles. Consistently, sample C5 is the most distant from the others in the PCA plot, reinforcing its classification as an outlier and justifying its exclusion from downstream analyses.

Figure S4. Modules of co-expressed genes in neurons are dysregulated in PMS. (A) Network analysis dendrogram showing clustering of genes based on topological overlap for identification of modules of co-regulated genes in iPSC-derived neurons. (B) Heatmap of correlation among modules, age, condition, sex and type of mutation. (C) Z-scores of preservations of identified modules in unrelated fetal brain modules derived from transcriptome data of BrainSpan curated samples.

Figure S5. Network of co-expressed genes correlated with PMS. Protein–protein interaction networks showing biological evidence of interaction of the top genes (kME > 0.9) assigned to each of the co-expression modules identified by WGCNA. Not connected nodes were hidden.

Figure S6. Gene co-expression modules and associated biological processes enriched in SHANK3-mutated (PMS) and control samples. (A–C) Bar plots showing the module eigengene (ME) values for each sample in the Lightgreen (A), Royalblue (B), and Turquoise (C) modules. Each bar represents an individual sample, with controls (C1–C7) shown in cyan and SHANK3-mutated samples (P1–P14, P2.2, P4–P9 and Ed1) in red. Positive and negative ME values indicate the relative expression level of the module's gene set in each sample. The lower panels display the top enriched Gene Ontology (GO) Biological Process (BP) terms for each module based on functional enrichment analysis. The x-axis indicates the $-\log_{10}$(p-value) of enrichment for each term. The vertical dashed red line marks the significance threshold ($p = 0.05$).

Figure S7. Relative mRNA expression of neural progenitors, excitatory and inhibitory neurons. Bar plots of relative mRNA expression of PAX6 (neural progenitor), GAD65 (inhibitory neurons), GAD67 (inhibitory neurons) and BCL11B (excitatory neurons). Induced pluripotent stem cells (iPSC) was used as a negative control.

Figure S8. Flow cytometry analysis of neural progenitors marker panel. A) Shown are gating of representative graphs for controls and SHANK3-mutated groups included in the statistical analysis of cell type proportions in cultures. Gates were defined using isotype antibody controls (red) as a reference. B) Bar plots of cell proportions. Mann-Whitney test *$p<0.05$; **$p<0.01$.

Figure S9. Flow cytometry analysis of proliferative progenitor markers. A) Gating and graphs of proportion of Edu and PAX6 cells. B) Gating and graphs of proportion of Edu and TBR2 positive cells. Shown are gating of representative graphs for controls and SHANK3-mutated groups included in the statistical analysis of cell type proportions in cultures. Gates were defined using isotype antibody controls (red) as a reference.

Figure S10. Cell cycle flow cytometry analysis. Graphic of cell cycle phases observed with flow cytometry in PMS and Controls neural cells population.

Figure S11. Flow cytometry analysis of DCX and GFAP markers. A) Gating and graphs of proportion of DCX positive cells. B) Gating and graphs of proportion of GFAP positive cells. Shown are gating of representative graphs for controls and SHANK3-mutated groups included in the statistical analysis of cell type proportions in cultures. Gates were defined using isotype antibody controls (red) as a reference. Mann-Whitney test *$p<0.05$; **$p<0.01$.

Figure S1.

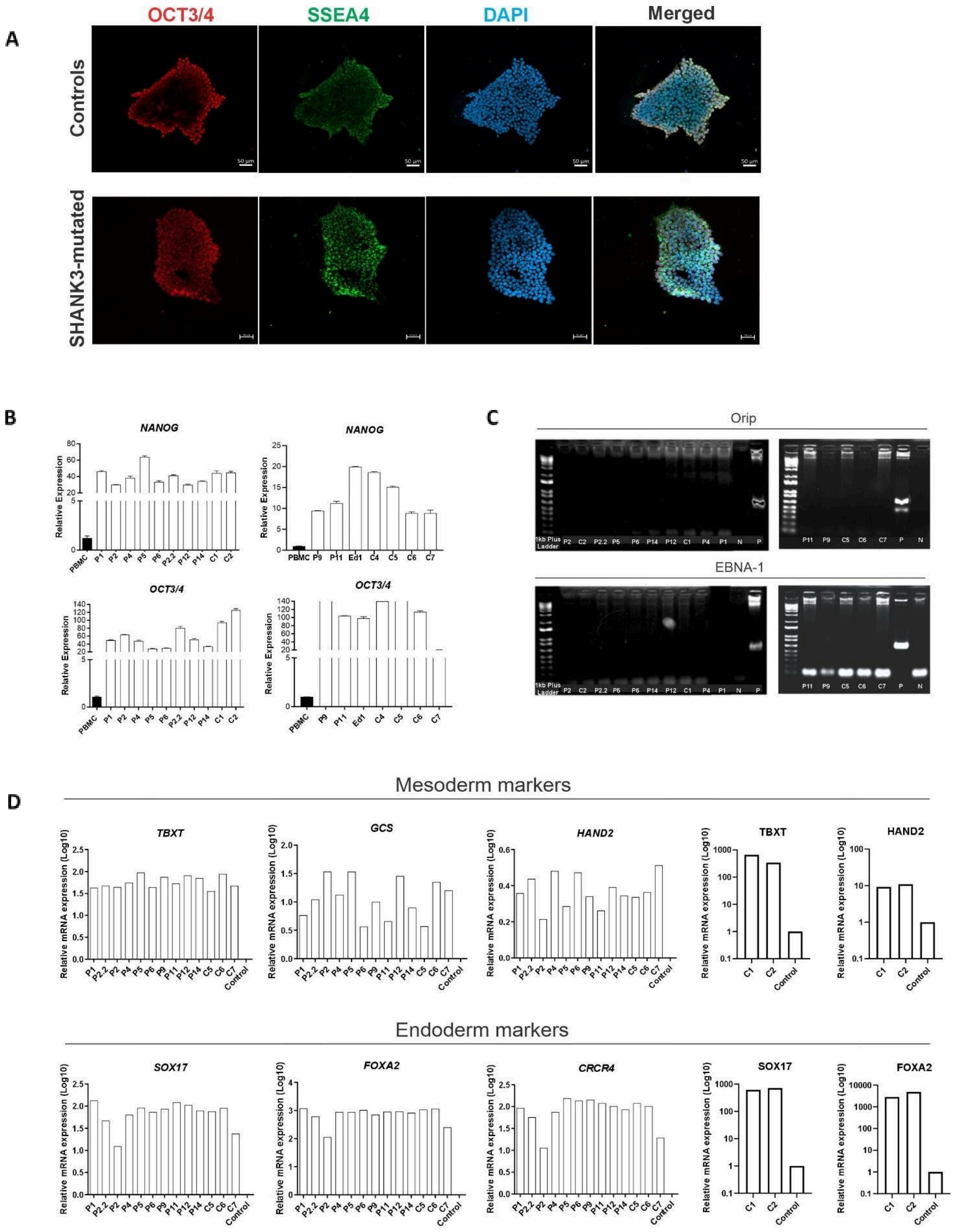
A
OCT3/4
SSEA4
DAPI
Merged
Controls
SHANK3-mutated
50 µm
B
NANOG
OCT3/4
Relative Expression
PBMC
P1
P2
P4
P5
P6
P2.2
P12
P14
C1
C2
P9
P11
Ed1
C4
C5
C6
C7
C
Orip
EBNA-1
1kb Plus Ladder
N
P
D
Mesoderm markers
TBXT
GCS
HAND2
Relative mRNA expression (Log10)
Control
Endoderm markers
SOX17
FOXA2
CRCR4

Figure S2.

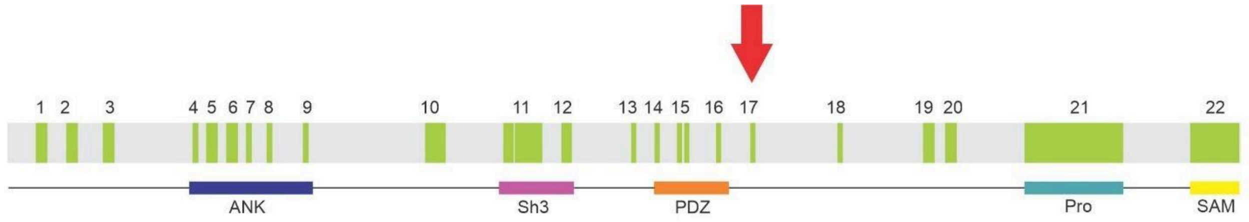

Status	Guide Target	PAM Sequence	Indel %	Model Fit (R^2)	Knockout-Score	Nuclease
Succeeded<br>WARNING – Padding alignment window to be at least 40 in length or to start of seq	ACACAACCTTCATGACGAGG	CGG	96	0.96	96	

POWERED BY SYNTHEGO ICE

RELATIVE CONTRIBUTION OF EACH SEQUENCE (NORMALIZED)

INDEL	CONTRIBUTION	SEQUENCE
-7	96%	TGTCACAGACACAACCTTCATGACG ¦ - - - - - - - TGCCACCCTGGCGAATCAGAGCCACCACCTGCTTGTGTCCGAC

The contributions show the inferred sequences present in your edited population and their relative proportions (in contrast to the Indel plot (Indel Distribution tab) that does not specify sequence contributions). Cut sites are represented by black vertical dotted lines, and the wild-type sequence is marked by a "+" symbol on the far left.

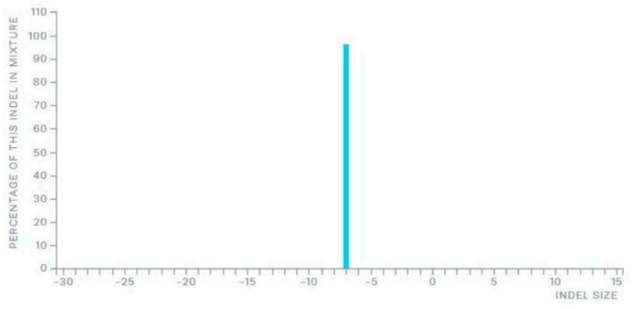

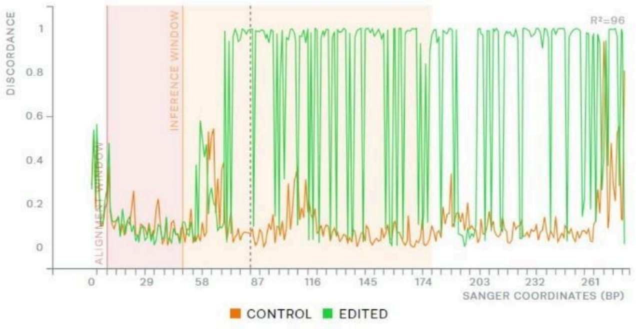

The Indel plot displays the inferred distribution of indels in the entire edited population of genomes. Hovering over each bar of the Indel plot shows the size of the insertion or deletion (+ or - 1 or more nucleotides), along with the percentage of genomes that contain it. Note: Each indel size represented in the Indel plot may not necessarily occur in the same sequence. The percentages of different indel sizes in the cell population are not the same as ICE/KO-Score scores.

The discordance plot details the level of alignment per base between the wild type (control) and the edited sample in the inference window (the region around the cute site), i.e. it shows the average amount of signal that disagrees with the reference sequence derived from the control trace file. On the plot, the green line and orange line should be close together before the cut site, with a typical CRISPR edit resulting in a jump in the discordance near the cutsite and continuing to remain far apart after the cut site (representing a high level of sequence discordance).

Figure S3.

A

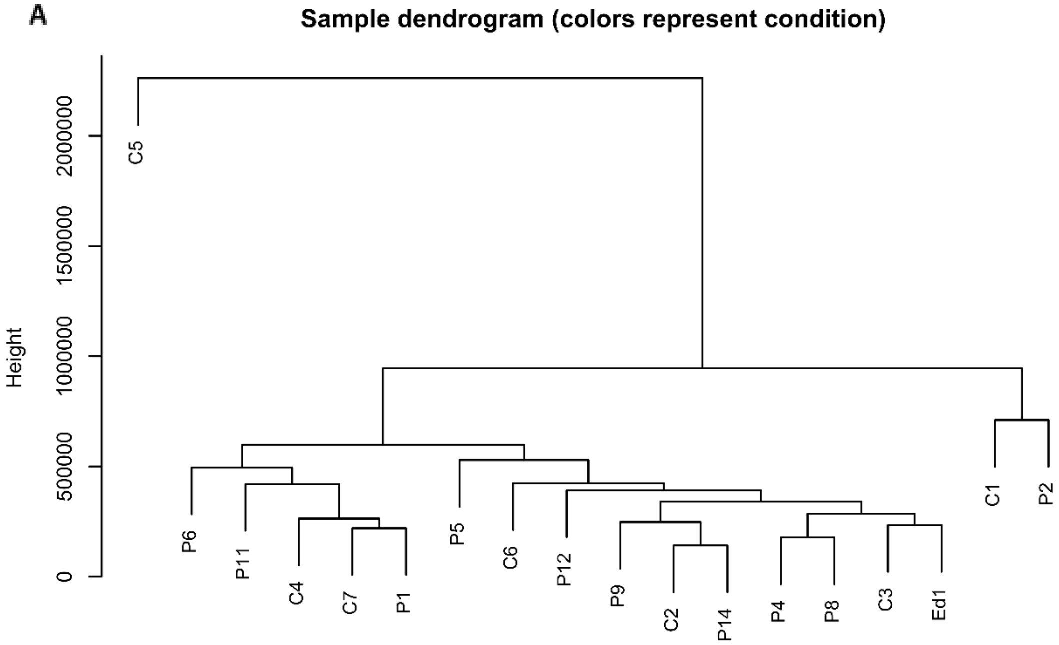

Sample dendrogram (colors represent condition)
Height
0
500000
1000000
1500000
2000000
C5
P6
P11
C4
C7
P1
P5
C6
P12
P9
C2
P14
P4
P8
C3
Ed1
C1
P2

B

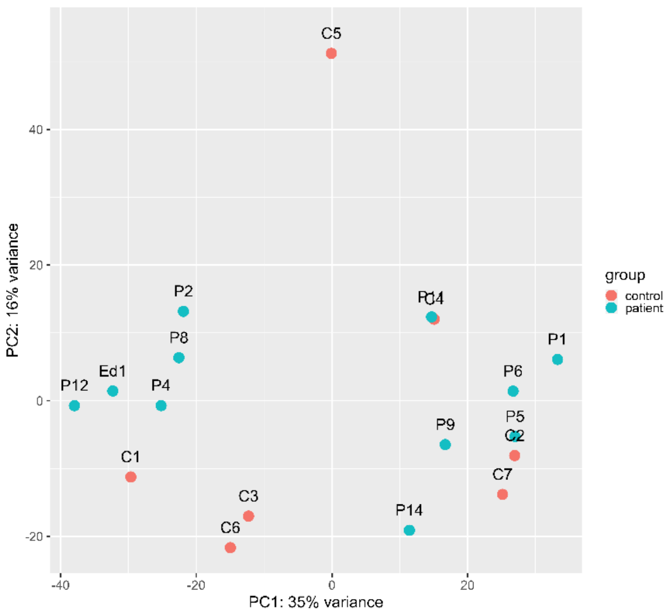

PC1: 35% variance
PC2: 16% variance
group
control
patient
C5
P2
P8
P12
Ed1
P4
C1
C3
C6
P14
P1
P6
P5
P9
C7

Figure S4.

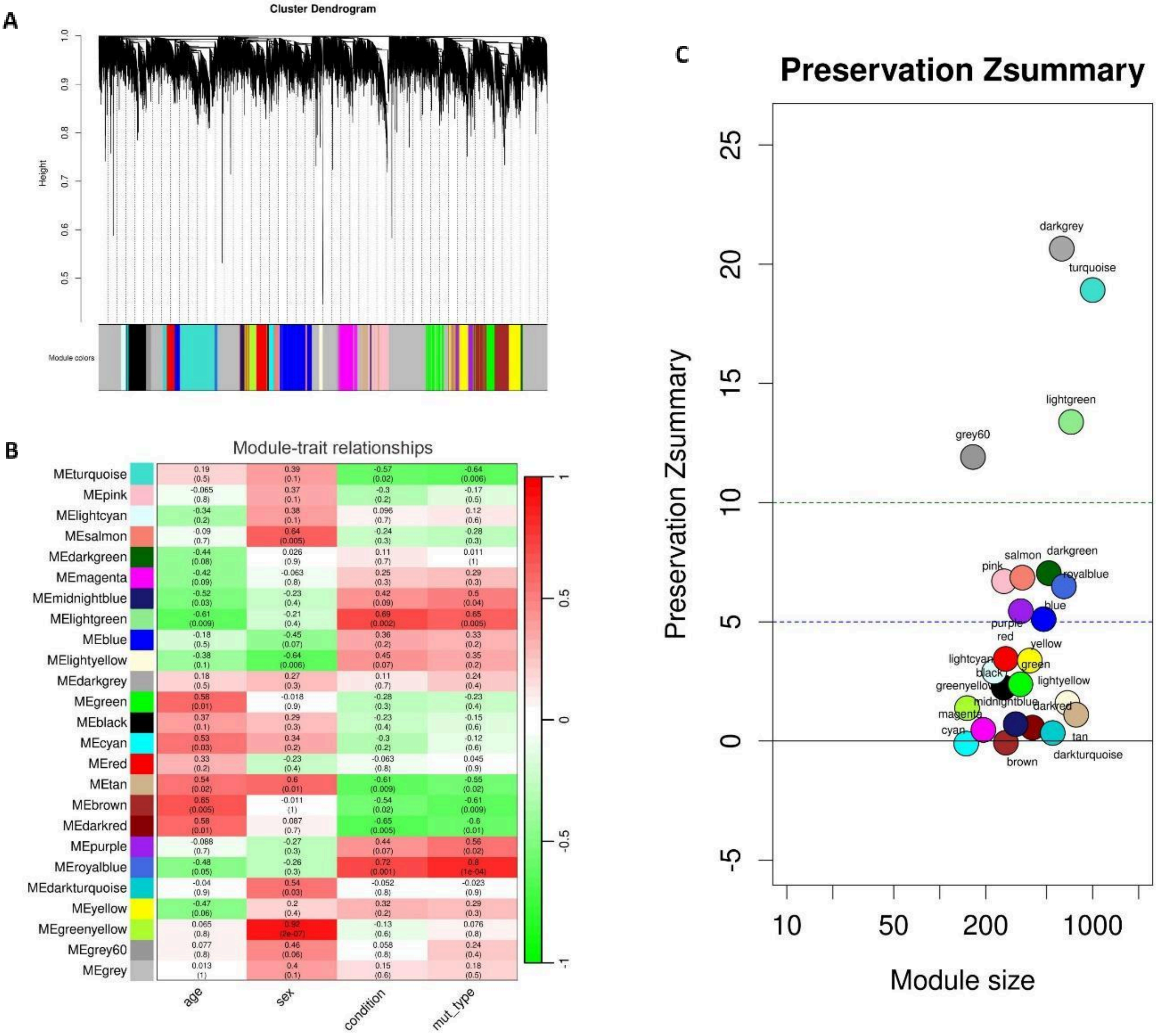
A
Cluster Dendrogram
Height
Module colors
B
Module-trait relationships
MEturquoise
MEpink
MElightcyan
MEsalmon
MEdarkgreen
MEmagenta
MEmidnightblue
MElightgreen
MEblue
MElightyellow
MEdarkgrey
MEgreen
MEblack
MEcyan
MEred
MEtan
MEbrown
MEdarkred
MEpurple
MEroyalblue
MEdarkturquoise
MEyellow
MEgreenyellow
MEgrey60
MEgrey
age
sex
condition
mut_type
C
Preservation Zsummary
Preservation Zsummary
Module size
darkgrey
turquoise
lightgreen
grey60
salmon
darkgreen
pink
royalblue
purple
blue
red
yellow
lightcyan
green
black
lightyellow
greenyellow
midnightblue
darkred
magenta
cyan
tan
brown
darkturquoise

Figure S5.

Lightgreen

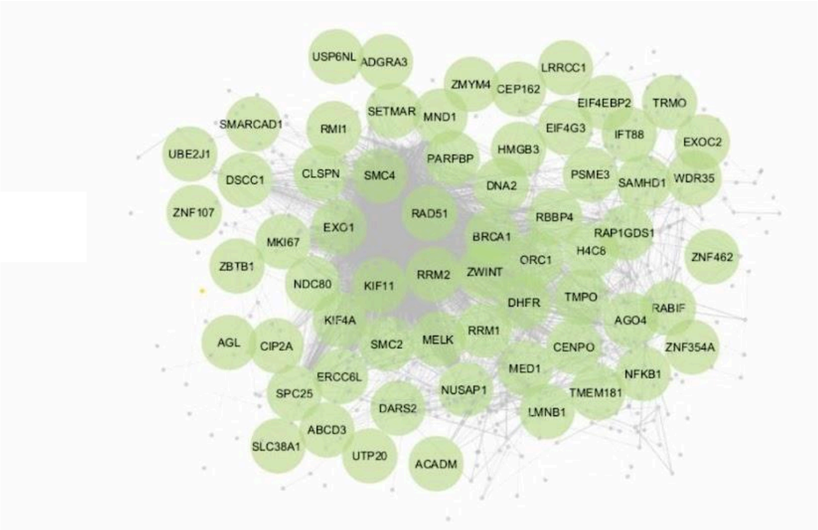

Royalblue

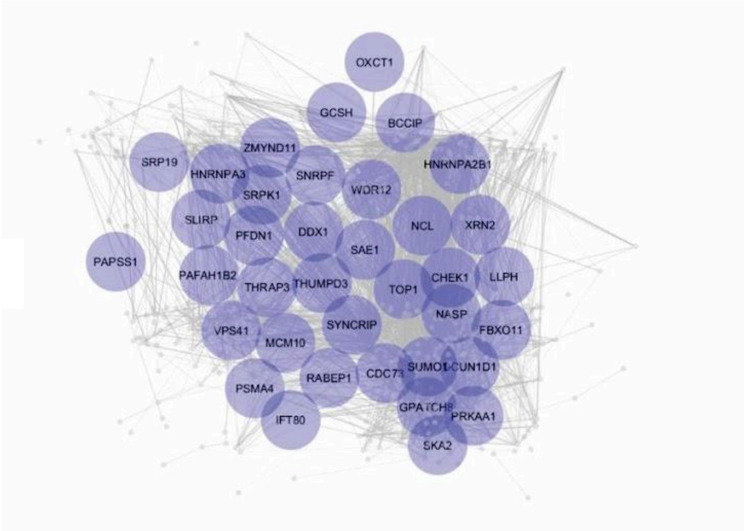

Turquoise

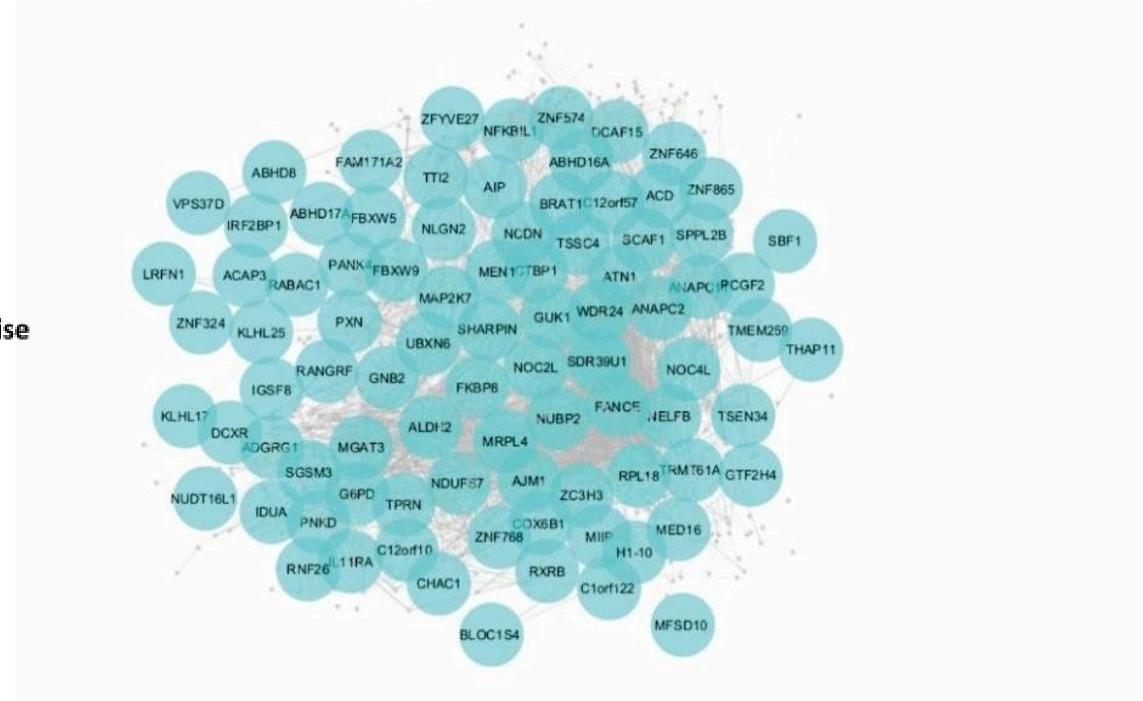

Figure S6.

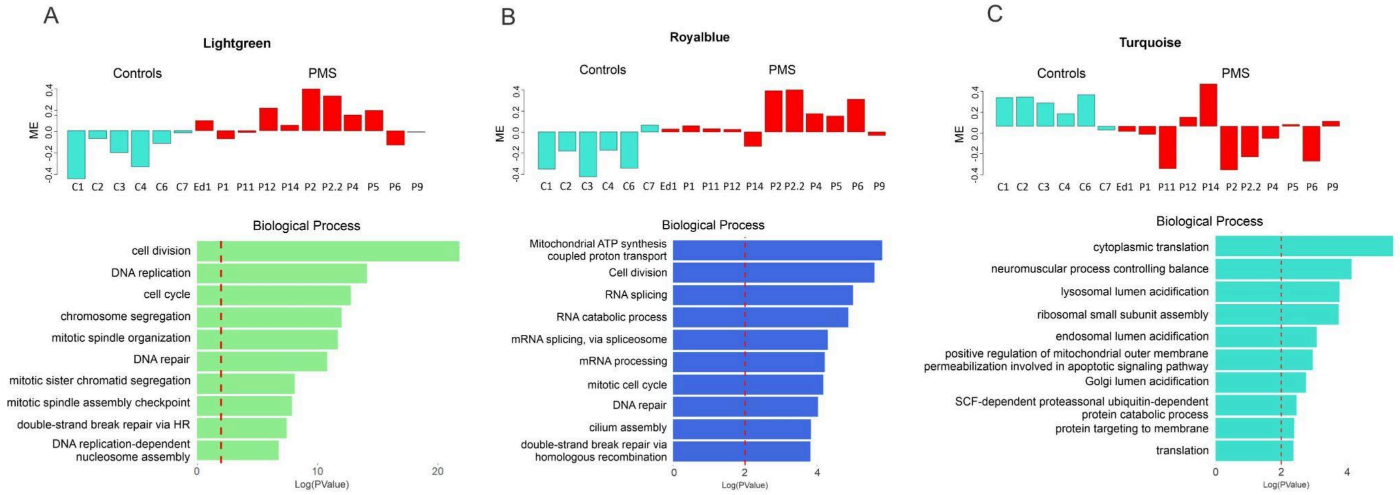

A
Lightgreen
Controls
PMS
ME
C1 C2 C3 C4 C6 C7 Ed1 P1 P11 P12 P14 P2 P2.2 P4 P5 P6 P9
Biological Process
cell division
DNA replication
cell cycle
chromosome segregation
mitotic spindle organization
DNA repair
mitotic sister chromatid segregation
mitotic spindle assembly checkpoint
double-strand break repair via HR
DNA replication-dependent nucleosome assembly
Log(PValue)
B
Royalblue
Controls
PMS
ME
Biological Process
Mitochondrial ATP synthesis coupled proton transport
Cell division
RNA splicing
RNA catabolic process
mRNA splicing, via spliceosome
mRNA processing
mitotic cell cycle
DNA repair
cilium assembly
double-strand break repair via homologous recombination
Log(PValue)
C
Turquoise
Controls
PMS
ME
Biological Process
cytoplasmic translation
neuromuscular process controlling balance
lysosomal lumen acidification
ribosomal small subunit assembly
endosomal lumen acidification
positive regulation of mitochondrial outer membrane permeabilization involved in apoptotic signaling pathway
Golgi lumen acidification
SCF-dependent proteasomal ubiquitin-dependent protein catabolic process
protein targeting to membrane
translation
Log(PValue)

Figure S7.

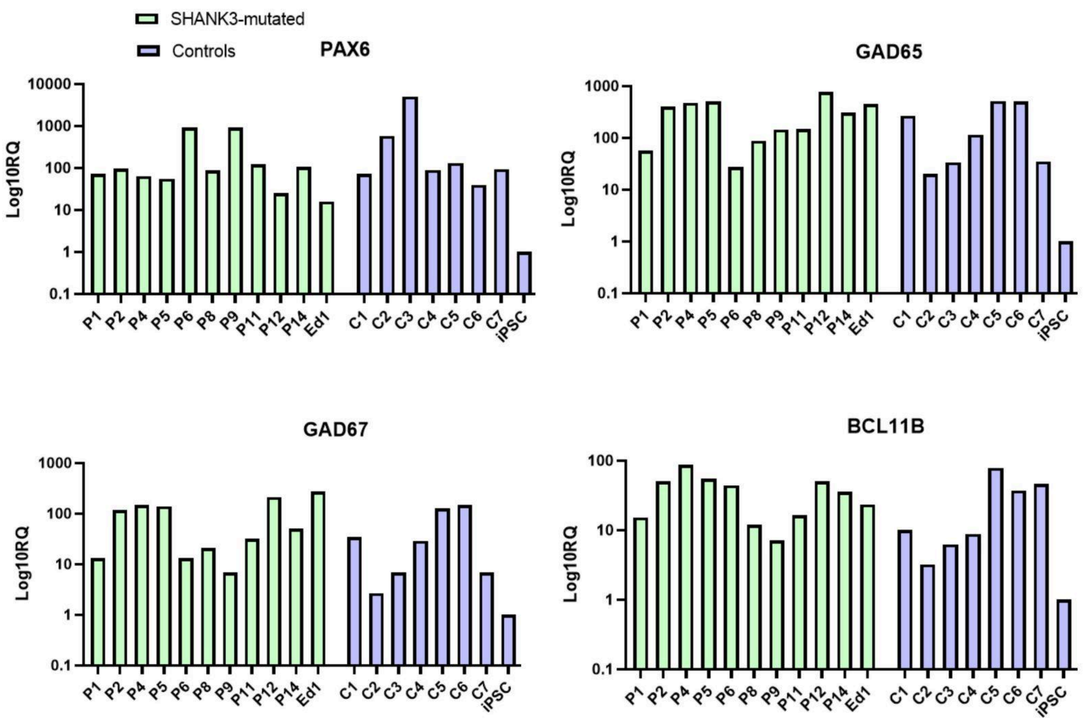
SHANK3-mutated
Controls
PAX6
GAD65
GAD67
BCL11B
Log10RQ
P1 P2 P4 P5 P6 P8 P9 P11 P12 P14 Ed1
C1 C2 C3 C4 C5 C6 C7 iPSC

Figure S8.

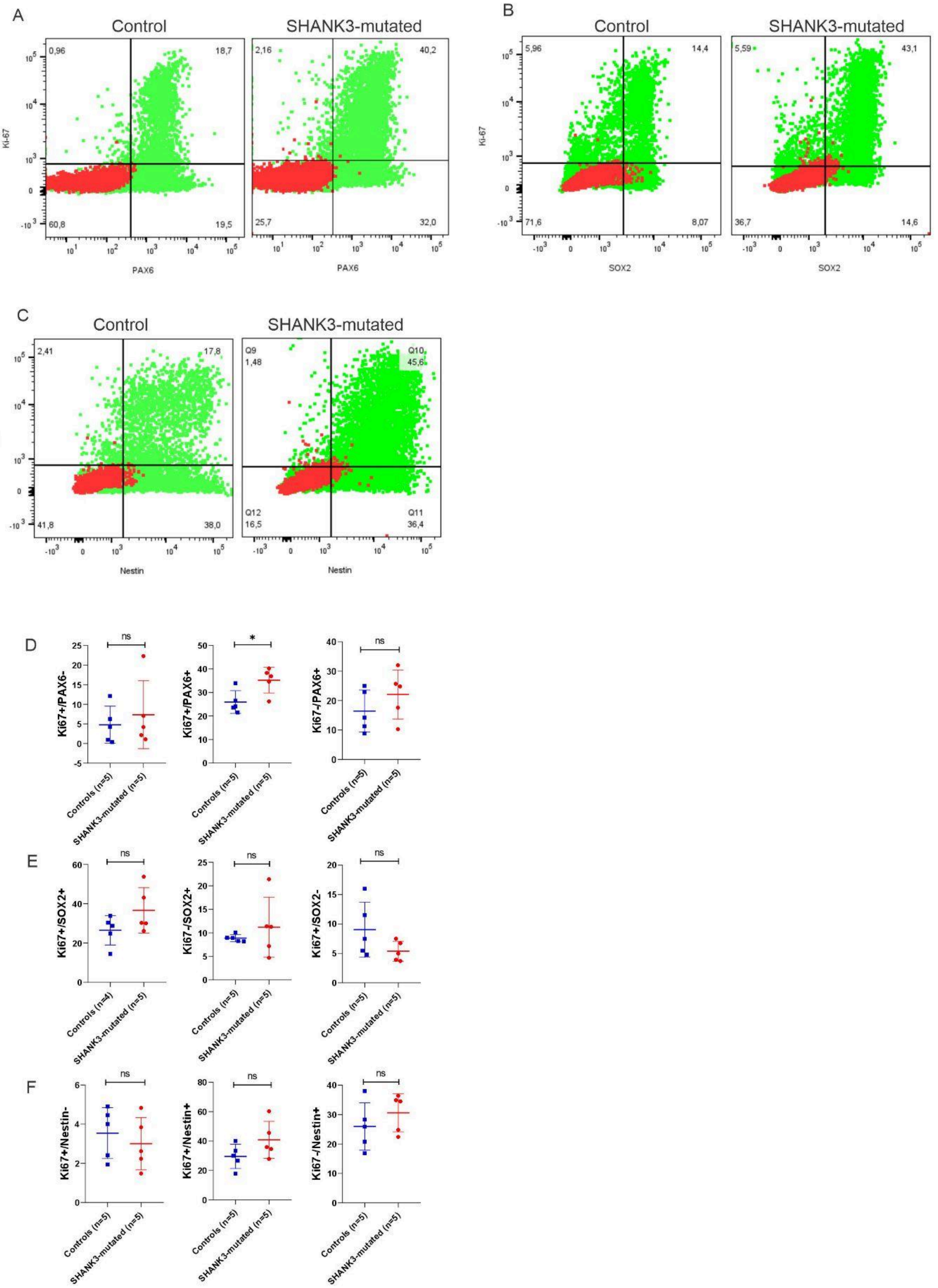

A
Control
SHANK3-mutated
0,96
18,7
60,8
19,5
2,16
40,2
25,7
32,0
Ki-67
PAX6
B
Control
SHANK3-mutated
5,96
14,4
71,6
8,07
5,59
43,1
36,7
14,6
Ki-67
SOX2
C
Control
SHANK3-mutated
2,41
17,8
41,8
38,0
Q9
1,48
Q10
45,6
Q12
16,5
Q11
36,4
Ki-67
Nestin
D
ns
Ki67+/PAX6-
*
Ki67+/PAX6+
ns
Ki67-/PAX6+
Controls (n=5)
SHANK3-mutated (n=5)
E
ns
Ki67+/SOX2+
Controls (n=4)
SHANK3-mutated (n=5)
ns
Ki67-/SOX2+
ns
Ki67+/SOX2-
Controls (n=5)
SHANK3-mutated (n=5)
F
ns
Ki67+/Nestin-
ns
Ki67+/Nestin+
ns
Ki67-/Nestin+
Controls (n=5)
SHANK3-mutated (n=5)

Figure S9.

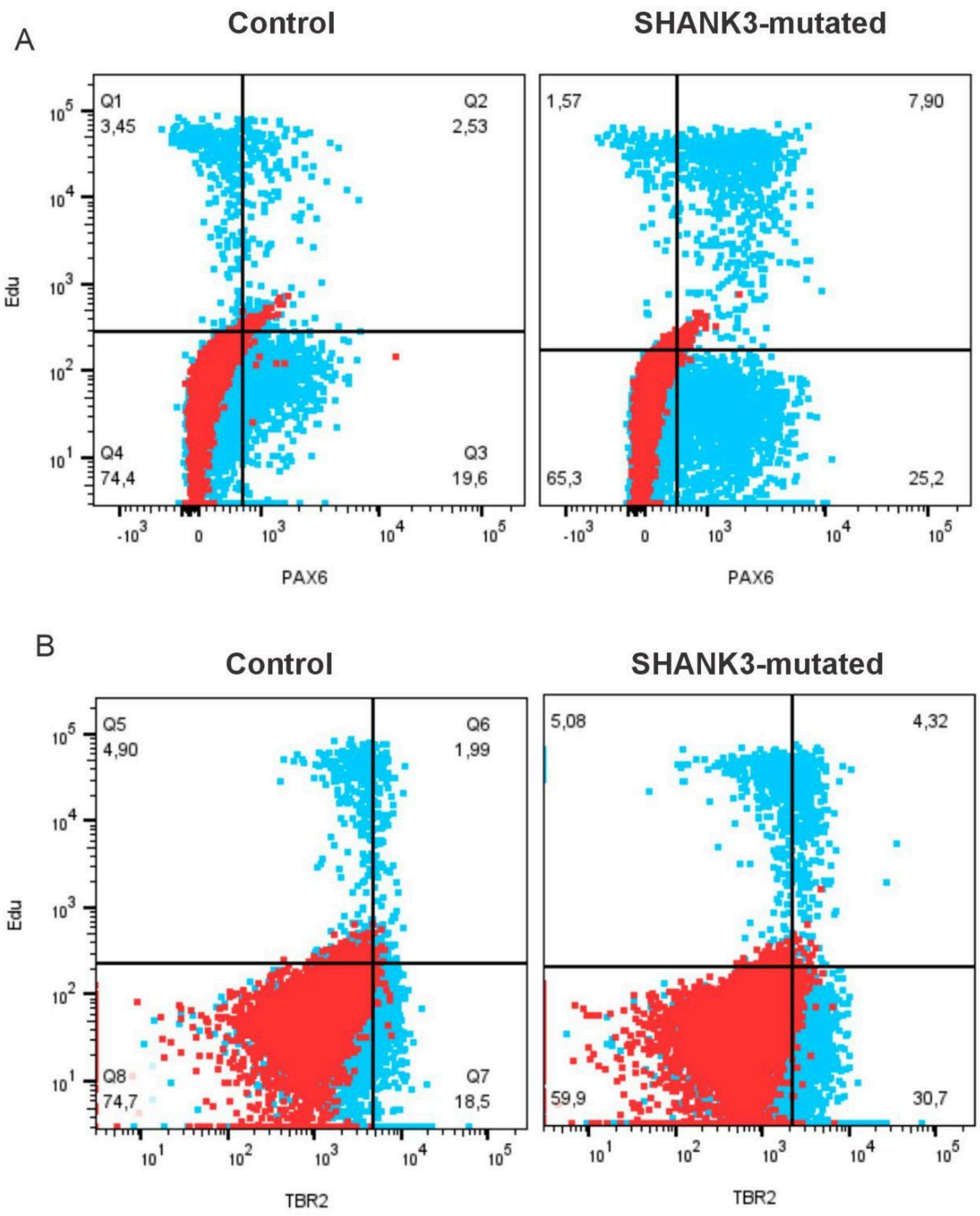
A
Control
SHANK3-mutated
Q1
3,45
Q2
2,53
Q4
74,4
Q3
19,6
1,57
7,90
65,3
25,2
Edu
PAX6
B
Control
SHANK3-mutated
Q5
4,90
Q6
1,99
Q8
74,7
Q7
18,5
5,08
4,32
59,9
30,7
Edu
TBR2

Figure S10.

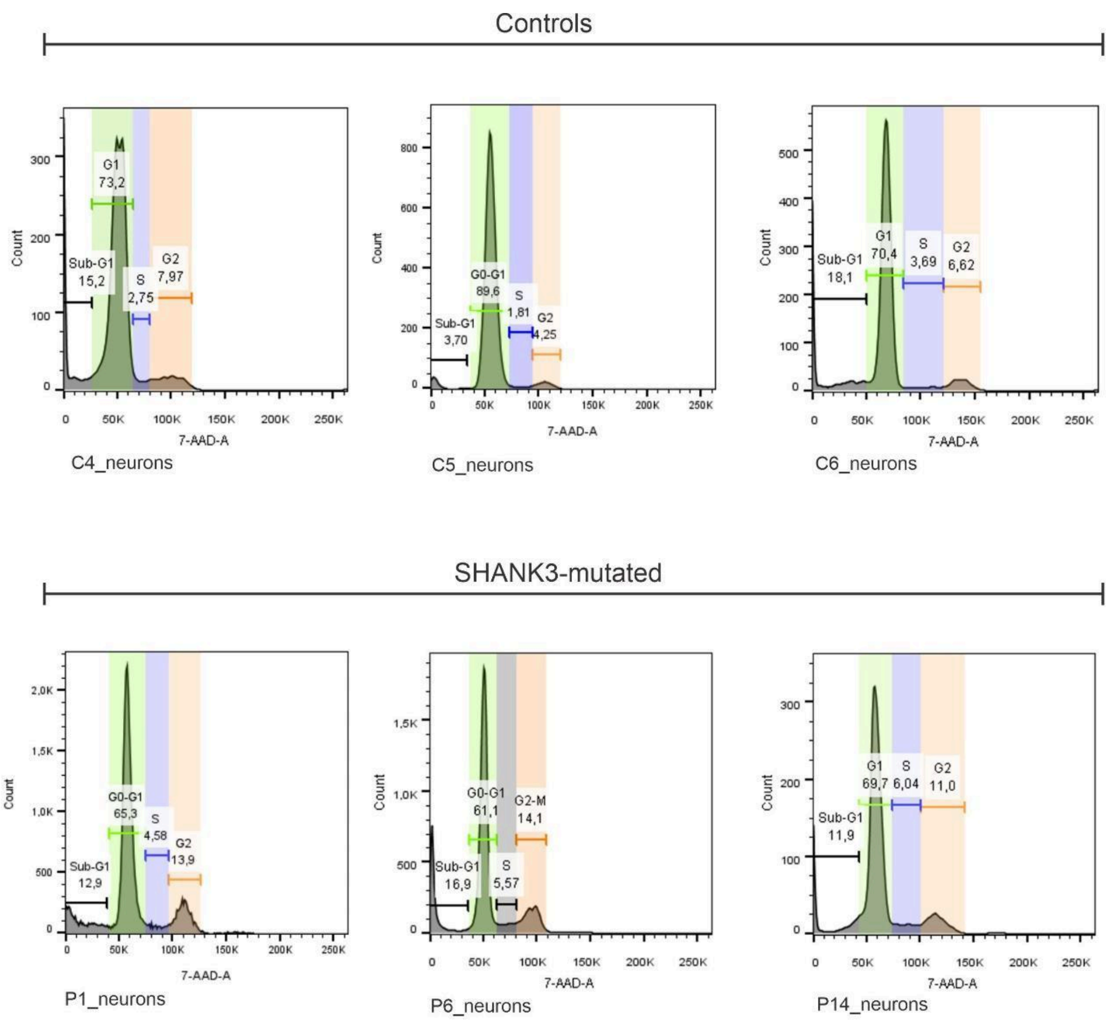
Controls
C4_neurons
G1 73,2
Sub-G1 15,2
S 2,75
G2 7,97
C5_neurons
G0-G1 89,6
Sub-G1 3,70
S 1,81
G2 4,25
C6_neurons
Sub-G1 18,1
G1 70,4
S 3,69
G2 6,62
SHANK3-mutated
P1_neurons
G0-G1 65,3
Sub-G1 12,9
S 4,58
G2 13,9
P6_neurons
G0-G1 61,1
G2-M 14,1
Sub-G1 16,9
S 5,57
P14_neurons
G1 69,7
S 6,04
G2 11,0
Sub-G1 11,9
Count
7-AAD-A

Figure S11.

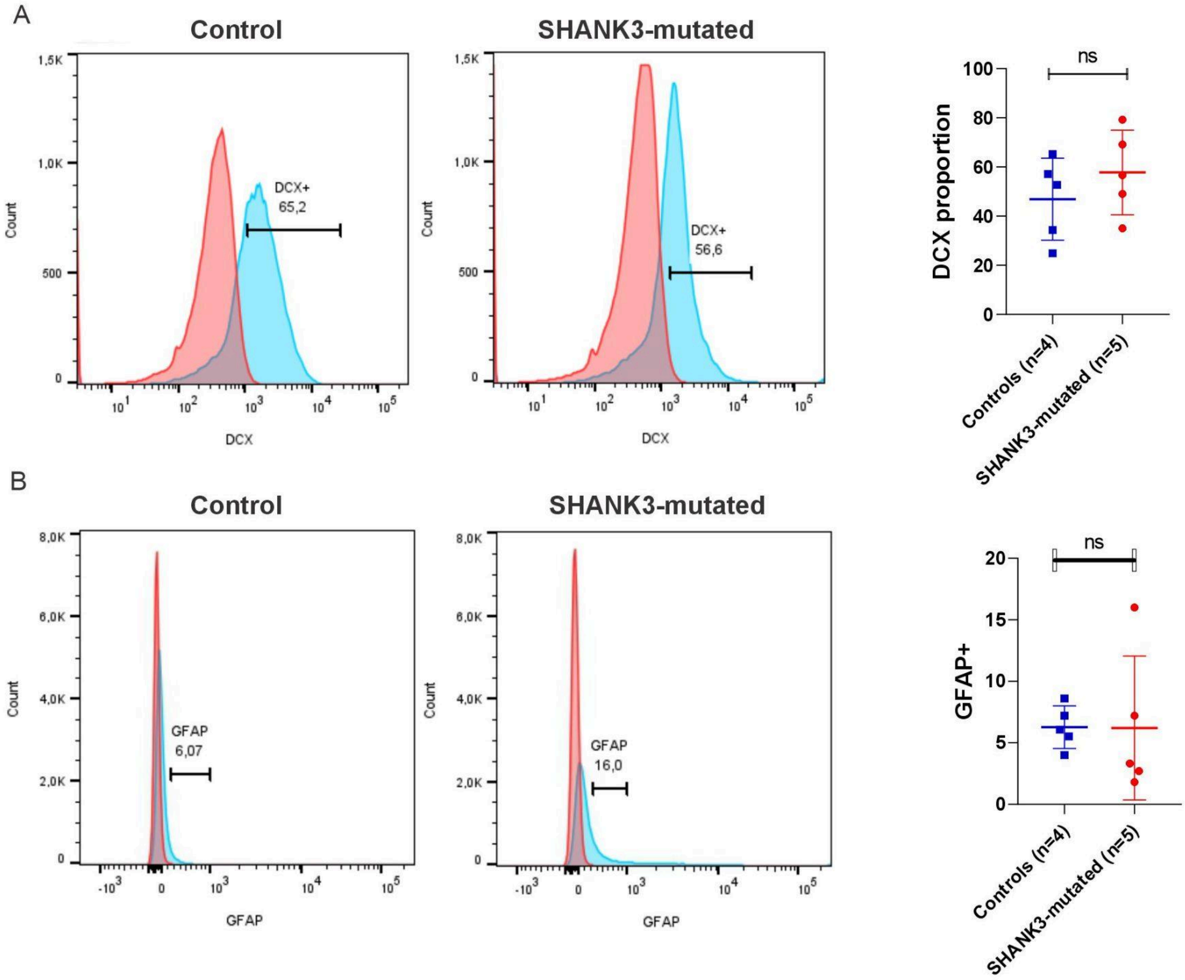
A
Control
SHANK3-mutated
Count
DCX
DCX+
65,2
DCX+
56,6
DCX proportion
ns
Controls (n=4)
SHANK3-mutated (n=5)
B
Control
SHANK3-mutated
Count
GFAP
GFAP
6,07
GFAP
16,0
GFAP+
ns
Controls (n=4)
SHANK3-mutated (n=5)

Supplementary Table

Table S2. Clones used by experiment

							Experiments						
**ID**	**Sex**	**Condition**	**Origin**	**Clone used**	**iPSC characterization**	**NSC IF markers**	**Neurons (MAP2 and TUJ1 expression)**	**RNA-seq**	**Morphological aspects**	**Synaptic puncta quantification**	**MEA**	**Cell cycle**	**Flow cytometry quantification**
P1	M	Patient	PBMC	cl1	RT-PCR; IF; MLPA; Array-CGH; 3 germ lines, OriP and STR	X	X	X	X	X	X	X	X
P2	F	Patient	PBMC	cl5	RT-PCR; IF; MLPA; Array-CGH; 3 germ lines, OriP and STR	X	X	X	X				
P2.2 (second clone of P2)	F	Patient	PBMC	cl6	RT-PCR; IF; MLPA; Array-CGH; 3 germ lines, OriP and STR		X	X		X			X
P4	F	Patient	PBMC	cl1	RT-PCR; IF; Array-CGH; 3 germ lines, OriP and STR	X	X	X	X	X			X
P5	M	Patient	PBMC	cl1	RT-PCR; IF; Array-CGH; 3 germ lines, OriP and STR	X	X	X			X		
P6	M	Patient	PBMC	cl2	RT-PCR; IF; Array-CGH; 3 germ lines, OriP and STR	X	X	X	X		X	X	X
P9	M	Patient	PBMC	cl1	RT-PCR; IF; Array-CGH; 3 germ lines, OriP and STR		X	X					
P11	F	Patient	PBMC	cl1	RT-PCR; IF; Array-CGH; 3 germ lines, OriP and STR	X	X	X	X		X		
P12	F	Patient	PBMC	cl1	RT-PCR; IF; Array-CGH; 3 germ lines, OriP and STR	X	X	X	X	X	X		
P14	M	Patient	PBMC	cl1	RT-PCR; IF; Array-CGH; 3 germ lines, OriP and STR	X	X	X	X		X	X	X
C1	F	Control	PBMC	cl1	RT-PCR; IF; MLPA; Array-CGH; 3 germ lines, OriP and STR	X	X	X	X	X	X		X
C2	M	Control	PBMC	cl1	RT-PCR; IF; MLPA; Array-CGH; 3 germ lines, OriP and STR	X	X	X	X	X			X
C3	M	Control	PBMC	cl1	Already published (doi: 10.3389/fncel.2021.803302)	X	X	X	X	X			X
C4	F	Control	PBMC	cl1	Already published (doi: 10.3389/fncel.2021.803302)	X	X	X	X	X	X	X	X
C5	F	trol (Mothe	PBMC	cl1	RT-PCR; IF; Array-CGH; 3 germ lines, OriP and STR	X	X	X (Low quality sequencing)			X	X	
C6	M	trol (Fathe	PBMC	cl3	RT-PCR; IF; Array-CGH; 3 germ lines, OriP and STR	X	X	X		X		X	X
C7	M	trol (Sibling	PBMC	cl3	RT-PCR; IF; Array-CGH; 3 germ lines, OriP and STR	X	X	X			X		
Ed1	F (from C4)	Isogenic	PBMC	cl1	RT-PCR; IF; 3 germ lines, OriP and STR		X	X	X	X			

Table S3. Samples used in RNAseq and quality parameters.

Sample name	Clone	Condition	ID run	Mapped sequences	Reads size STAR
C1	cl1	Control	EVB-10	63170165	267
C2	cl1	Control	EVB-11	131170601	275
C3	cl1	Control	EVB-12	68153698	278
C4	cl1	Control	EVB-13	65134805	277
C5	cl1	Control	EVB-14	112996322	278
C6	cl3	Control	EVB-15	208758203	281
C7	cl3	Control	EVB-16	89685453	287
Ed1	cl1	Isogenic	EVB-35	86124624	283
P1	cl1	Patient	EVB-1	81180701	285
P11	cl1	Patient	EVB-7	60433216	281
P12	cl1	Patient	EVB-8	69169739	282
P14	cl1	Patient	EVB-9	175547894	282
P2	cl5	Patient	EVB-19	113449676	286
P2.2	cl6	Patient	EVB-23	197548886	290
P4	cl1	Patient	EVB-20	109388130	282
P5	cl1	Patient	EVB-21	194871052	285
P6	cl2	Patient	EVB-22	128637072	279
P9	cl1	Patient	EVB-6	65601137	279

Table S8. Descriptive statistics of EEG cohort include in this study.

	Group	% good electrodes	Segments number	theta (4-7.5)	alpha (8-12)	beta (13-20)	gamma (30-45)	SCQ	Vineland	Age (Months)
**N**	Control	26	26	27	27	27	27	19	20	29
	Patient	18	18	18	18	18	18	16	17	22
**Missing**	Control	3	3	2	2	2	2	10	9	0
	Patient	4	4	4	4	4	4	6	5	0
**Average**	Control	86.1	115	0.00415	0.00904	0.00614	0.0188	4.68	101	108
	Patient	93.6	93.1	0.00516	0.00753	0.00734	0.0325	19.5	45.6	99.9
**Median**	Control	87.2	117	0.00383	0.00630	0.00472	0.0149	5	101	97
	Patient	95.4	107	0.00184	0.00322	0.00540	0.0223	20.5	44	98.0
**Standard deviation**	Control	8.68	27.9	0.00386	0.00883	0.00636	0.0152	2.52	13.5	64.3
	Patient	4.85	46.3	0.00799	0.0108	0.00903	0.0311	6.31	14.7	58.9
**Minimum**	Control	72.5	25	-1.17e-4	-1.00e-4	-6.38e-5	0.00308	1	83	15
	Patient	81.6	16	-0.00212	-0.00401	-0.00143	5.73E-4	8	23	12
**Maximum**	Control	100	184	0.0148	0.0305	0.0312	0.0616	9	133	235
	Patient	99.0	147	0.0254	0.0416	0.0312	0.101	27	75	218
